# Low-level blast exposure disrupts gliovascular and neurovascular connections and induces a chronic vascular pathology in rat brain

**DOI:** 10.1186/s40478-018-0647-5

**Published:** 2019-01-09

**Authors:** Miguel A. Gama Sosa, Rita De Gasperi, Georgina S. Perez Garcia, Gissel M. Perez, Courtney Searcy, Danielle Vargas, Alicia Spencer, Pierce L. Janssen, Anna E. Tschiffely, Richard M. McCarron, Benjamin Ache, Rajaram Manoharan, William G. Janssen, Susan J. Tappan, Russell W. Hanson, Sam Gandy, Patrick R. Hof, Stephen T. Ahlers, Gregory A. Elder

**Affiliations:** 1 General Medical Research Service, James J. Peters Department of Veterans Affairs Medical Center, 130 West Kingsbridge Road, Bronx, NY 10468 USA; 2 Department of Psychiatry, Icahn School of Medicine at Mount Sinai, One Gustave Levy Place, New York, NY 10029 USA; 3 Friedman Brain Institute, Icahn School of Medicine at Mount Sinai, New York, NY 10029 USA; 4 Research and Development Service, James J. Peters Department of Veterans Affairs Medical Center, 130 West Kingsbridge Road, Bronx, NY 10468 USA; 5 Department of Neurology, Icahn School of Medicine at Mount Sinai, One Gustave Levy Place, New York, NY 10029 USA; 6 Fishberg Department of Neuroscience, Icahn School of Medicine at Mount Sinai, New York, NY 10029 USA; 7 Department of Neurotrauma, Operational and Undersea Medicine Directorate, Naval Medical Research Center, 503 Robert Grant Avenue, Silver Spring, MD 20910 USA; 8 Department of Surgery, Uniformed Services University of the Health Sciences, Bethesda, MD 20914 USA; 9 Micro Photonics, Inc., 1550 Pond Road, Allentown, PA 18104 USA; 10 MBF Bioscience, 185 Allen Brook Lane, Williston, VT 05495 USA; 11 Department of Genetics and Genomic Sciences, Icahn School of Medicine at Mount Sinai, New York, NY 10029 USA; 12 Mount Sinai Alzheimer’s Disease Research Center and the Ronald M. Loeb Center for Alzheimer’s Disease, Icahn School of Medicine at Mount Sinai, New York, NY 10029 USA; 13 NFL Neurological Care Center, Icahn School of Medicine at Mount Sinai, New York, NY 10029 USA; 14 Department of Geriatrics and Palliative Care, Icahn School of Medicine at Mount Sinai, New York, NY 10029 USA; 15 Neurology Service, James J. Peters Department of Veterans Affairs Medical Center, 130 West Kingsbridge Road, Bronx, NY 10468 USA

**Keywords:** Animal model, Blast, Brain, Chronic, Gliovascular, Neurovascular, Rat, Vascular pathology

## Abstract

Much concern exists over the role of blast-induced traumatic brain injury (TBI) in the chronic cognitive and mental health problems that develop in veterans and active duty military personnel. The brain vasculature is particularly sensitive to blast injury. The aim of this study was to characterize the evolving molecular and histologic alterations in the neurovascular unit induced by three repetitive low-energy blast exposures (3 × 74.5 kPa) in a rat model mimicking human mild TBI or subclinical blast exposure. High-resolution two-dimensional differential gel electrophoresis (2D-DIGE) and matrix-assisted laser desorption/ionization (MALDI) mass spectrometry of purified brain vascular fractions from blast-exposed animals 6 weeks post-exposure showed decreased levels of vascular-associated glial fibrillary acidic protein (GFAP) and several neuronal intermediate filament proteins (α-internexin and the low, middle, and high molecular weight neurofilament subunits). Loss of these proteins suggested that blast exposure disrupts gliovascular and neurovascular interactions. Electron microscopy confirmed blast-induced effects on perivascular astrocytes including swelling and degeneration of astrocytic endfeet in the brain cortical vasculature. Because the astrocyte is a major sensor of neuronal activity and regulator of cerebral blood flow, structural disruption of gliovascular integrity within the neurovascular unit should impair cerebral autoregulation. Disrupted neurovascular connections to pial and parenchymal blood vessels might also affect brain circulation. Blast exposures also induced structural and functional alterations in the arterial smooth muscle layer. Interestingly, by 8 months after blast exposure, GFAP and neuronal intermediate filament expression had recovered to control levels in isolated brain vascular fractions. However, despite this recovery, a widespread vascular pathology was still apparent at 10 months after blast exposure histologically and on micro-computed tomography scanning. Thus, low-level blast exposure disrupts gliovascular and neurovascular connections while inducing a chronic vascular pathology.

## Introduction

Traumatic brain injury (TBI) has long been a major cause of combat-related disability [25]. Public awareness of TBI in the military has increased recently due to events in Iraq and Afghanistan where 10–20% of veterans returning from these conflicts experienced a TBI [28]. While military related TBIs in Iraq and Afghanistan resulted from various mechanisms due to the wide spread use of improvised explosive devices, blast-related TBIs were most common [28]. In humans high-pressure blast waves can cause extensive multi-organ trauma including severe systemic vascular and CNS injury [78, 91]. However, in combat settings such as Iraq and Afghanistan, lower level exposures producing mTBIs have been much more common [28]. While many veterans who suffered blast-related TBIs improve others exhibit chronic postconcussive and mental health related symptoms that are largely refractory to therapy [25, 50]. TBI, in particular repetitive mTBI, has also been associated with the later development of neurodegenerative diseases [23, 25, 35]. In addition there is increasing concern over the potential adverse consequences of subclinical blast exposures, which are common for many service members in non-combat settings [12].

How blast damages the nervous system is incompletely understood. The high metabolic demand of the brain requires a tight coordination between neuronal activity and blood flow [40]. Blast injury is known to affect the cerebral vasculature [27]. High level blast exposure induces prominent vasospasm in humans [5] and animals [8] along with reduced cerebral perfusion and altered contractile properties of large arteries [9, 73, 86]. As in humans, acute high-level blast exposure has a prominent hemorrhagic component which in animals includes venous hemorrhages [7, 27]. Blast-induced vasospasm has been suggested to in addition initiate a phenotypic switch in vascular smooth muscle cells that causes long term vascular remodeling [4, 39].

Many studies have described blast-related vascular pathology [1, 7, 18, 19, 27, 31, 32, 37, 42, 46–48, 51–53, 55, 58, 61, 68, 69, 72, 73, 76, 80, 84, 87]. At the functional level, acute blast exposure has been associated with increased vascular permeability and blood–brain barrier (BBB) breakdown. Multiple studies have described increases in BBB permeability as judged by leakage of IgG, Evans Blue or sodium-fluorescein low-molecular-weight tracers [1, 36, 45, 49, 52, 54, 56, 57, 60, 61, 71, 74, 81, 83, 85, 90, 92, 93]. Acutely, blast exposure in mice produces microlesions in the BBB that are associated with aberrant expression of phosphorylated tau protein [42, 60]. Much evidence also supports a mechanism whereby a blast wave striking the body causes indirect central nervous system injury through what has been referred to as a thoracic effect [13, 21, 27, 80].

The pathophysiological basis of blast-related vascular pathology remains incompletely understood. Morphological and functional data indicate that both large and small brain vessels are affected [27]. However, little is known about the molecular changes associated with these abnormalities. We have been studying a rat model of blast overpressure injury that mimics a repetitive low-level blast exposure similar to that which would be encountered in a human mTBI or subclinical blast exposure [2]. Under the conditions of exposure in our model, at the histological level the cerebral vasculature appears selectively vulnerable [31]. Here we show that blast injury disrupts gliovascular and neurovascular connections and is associated with a chronic vascular pathology. Because neuronal and astrocytic mechanisms control cerebral blood flow, disruption of gliovascular and neurovascular interactions should affect cerebral autoregulation at multiple levels.

## Material and methods

### Animals

Adult male Long Evans hooded rats (250–350 g, 10 weeks of age; Charles River Laboratories International, Wilmington, MA, USA) were used. All studies involving animals were reviewed and approved by the Institutional Animal Care and Use Committees of the Walter Reed Army Institute of Research (WRAIR)/Naval Medical Research Center and the James J. Peters VA Medical Center. Studies were conducted in compliance with the Public Health Service policy on the humane care and use of laboratory animals, the NIH Guide for the Care and Use of Laboratory Animals, and all applicable Federal regulations governing the protection of animals in research.

### Blast overpressure exposure

Rats were exposed to overpressure injury using the WRAIR shock tube, which simulates the effects of air blast exposure under experimental conditions. The shock tube has a 12-in. circular diameter and is a 19.5 ft. long steel tube divided into a 2.5 ft. compression chamber that is separated from a 17 ft. expansion chamber. The compression and expansion chambers are separated by polyethylene Mylar ™ sheets (Du Pont Co., Wilmington, DE, USA) that control the peak pressure generated. The peak pressure at the end of the expansion chamber was determined by piezoresistive gauges specifically designed for pressure-time (impulse) measurements (Model 102 M152, PCB, Piezotronics, Inc., Depew, NY, USA). This apparatus has been used in multiple prior studies to deliver blast overpressure injury to rats [2, 15–17, 22, 26, 31–33, 38, 64–66].

Individual rats were anesthetized using an isoflurane gas anesthesia system consisting of a vaporizer, gas lines and valves and an activated charcoal scavenging system adapted for use with rodents. Rats were placed into a polycarbonate induction chamber, which was closed and immediately flushed with 5% isoflurane in air mixture for 2 min. Rats were placed into a cone shaped plastic restraint device and then placed in the shock tube. Movement was further restricted during the blast exposure using 1.5 cm diameter flattened rubber tourniquet tubing. Three tourniquets were spaced evenly to secure the head region, the upper torso and lower torso while the animal was in the plastic restraint cone. The end of each tubing was threaded through a toggle and run outside of the exposure cage where it was tied to firmly affix the animal and prevent movement during the blast overpressure exposure without restricting breathing. Rats were randomly assigned to sham or blast conditions and were placed in the shock tube lying prone with the plane representing a line from the tail to the nose of the body in line with the longitudinal axis of the shock tube with the head placed more upstream. Further details of the physical characteristics of the blast wave are described in Ahlers et al. [2]. Blast exposed animals received 74.5 kPa (equivalent to 10.8 psi, duration 4.8 ms, impulse 175.8 kPa*ms) exposures administered one exposure per day for three consecutive days. Sham exposed animals were treated identically including receiving anesthesia and being placed in the blast tube but did not receive a blast exposure. Within 10 days after the last blast or sham exposure animals were transported in a climate controlled van from the WRAIR to the James J. Peters VA Medical Center (Bronx, NY, USA). Animals left in the morning from the WRAIR and arrived in the afternoon of the same day at the James J. Peters VA Medical Center, where all other procedures were performed.

### Brain vascular isolation

Rats were euthanized by CO_2_ inhalation, and the brain immediately dissected. Whole brains were cleaned of meninges and homogenized in cold 18% dextran in phosphate-buffered saline (PBS, 10 ml/g of tissue) using a Potter-Elvejehm homogenizer with a loose-fit Teflon pestle (6–8 strokes at low speed, setting 2 of a Wheaton overhead stirrer, Millville, NJ, USA). Homogenization resulted in a thick homogenate of low density that was overlayed over an equal volume of Ficoll-Paque PLUS™ (GE Healthcare Life Sciences, Marlborough, MA, USA) to form a single-step discontinuous gradient. Centrifugation was performed for 30 min at 1500×g and 4 °C. Pellets were then resuspended, washed twice with PBS and stored at − 80 °C for biochemical analyses or fixed in 4% paraformaldehyde (PFA) in PBS and kept at 4 °C until use.

### High-resolution two-dimensional differential gel electrophoresis

Purified vascular preparations from brains of control and blast-exposed rats (two samples/group) isolated from control and blast exposed brains 6 weeks after exposure were compared by two-dimensional differential gel electrophoresis (2D-DIGE). 2D-DIGE and subsequent identification of candidate proteins by matrix-assisted laser desorption/ionization (MALDI) mass spectrometry (MS) were performed by Applied Biomics, Inc., (Hayward, CA, USA). Samples were extracted with 2D lysis buffer (30 mM Tris HCl, pH 8.8, 7 M urea, 2 M thiourea, 4% CHAPS,). Control and blast samples were labeled with Cy2 and Cy5 dyes, respectively, and mixed with 2X 2D sample buffer (8 M urea, 4% CHAPS, 20 mg/ml DTT, 2% pharmalytes and trace bromophenol blue). DeStreak solution and rehydration buffer (7 M urea, 2 M thiourea, 4% CHAPS, 20 mg/ml DTT, 1% pharmalytes and 0.01% bromophenol blue) were added. Proteins were separated by isoelectric focusing (IEF) (3–10) followed by sodium dodecyl sulfate (SDS)-polyacrylamide gel electrophoresis (PAGE). Gels were imaged with a Typhoon Trio Imager (GE Healthcare Life Sciences) and analyzed with the Image QuantTL software (GE Healthcare Life Sciences). Gel and cross-gel analyses were performed using DeCyder software (GE Healthcare Life Sciences) to calculate the fold-change in protein expression levels between control and blast samples.

### Protein identification by MS

Candidates for protein identification were selected based on fold-change comparisons between control and blast samples and the calculated *p*-value. Spots of interest were selected using the Ettan Spot Picker (GE Healthcare) and in-gel digested with Trypsin Gold (Promega, Madison, WI, USA). The tryptic peptides were desalted with ZipTip C18 (Millipore, Billerica, MA, USA) and eluted with 5 mg/ml α-cyano-4-hydroxycinnamic acid in 25 mM ammonium bicarbonate, 50% acetonitrile and 0.1% trifluoroacetic acid and spotted onto an AB SCIEX MALDI plate (AB Sciex, Framingham, MA, USA). MALDI-TOF MS and TOF/TOF (tandem MS/MS) were performed using a 5800 mass spectrometer (AB Sciex, Concord, Ontario, Canada). The peptide mass and associated fragmentation data were determined using the GPS Explorer Workstation equipped with a MASCOT search engine (Matrix Science, Boston, MA, USA). Searches were performed without constraining protein molecular weight or isoelectric point, with variable methionine oxidation and cysteine carbamidomethylation, and with one missed cleavage permitted. Candidates with an ion confidence interval (CI%) or protein score CI% > 95 were considered significant.

### Western blot analysis

Vascular fractions were lysed in 10 mM NaPO_4_, pH 7.4, 150 mM NaCl, 2 mM EDTA, 1% Triton X-100, 0.5% sodium deoxycholate, and 1% SDS supplemented with protease and phosphatase inhibitor cocktails 2 and 3 (Sigma-Aldrich, St Louis, MO, USA). The lysates were centrifuged at 15000×g for 15 min, and the protein concentration in the supernatants was determined with the BCA reagent (ThermoFisher, Waltham MA, USA) according to the manufacturer’s protocol. Proteins (10–20 μg) were separated by SDS-PAGE, and the gels blotted onto polyvinylidene difluoride (PVDF) membranes. The membranes were blocked in a solution containing 50 mM Tris-HCl, pH 7.6, 0.15 M NaCl (TBS) and 0.5% non-fat dry milk and incubated overnight at 4 °C with primary antibody diluted in blocking solution. Membranes were incubated with the appropriate horseradish peroxidase-conjugated secondary antibody (1:5000–1:10,000, GE Healthcare Life Sciences) in blocking solution, and the bands visualized with the ECL Prime Western Blot detection reagent (GE Healthcare Life Sciences). The blots were imaged with the Amersham Imager 600 (GE Healthcare Life Sciences), and bands quantitated with Image QuantTL software (GE Healthcare Life Sciences). For analysis of GFAP expression, brain subregions were dissected from control and blast-exposed animals. Tissue lysates were analyzed as above except that 50 μg of protein were loaded onto the gel.

The following primary antibodies and dilutions were used: rabbit polyclonal anti-glial fibrillary acidic protein (anti-GFAP, 1:2000, G9269), rabbit polyclonal anti-neurofilament heavy polypeptide (anti-NFH, 1:2000, N4142) and mouse monoclonal anti-α-SMA (1:2000, α-smooth muscle actin clone 1A4, A25470; all from Sigma-Aldrich), rabbit polyclonal anti-α-internexin (anti–α-INX, 1:1000, GTX130053) and rabbit polyclonal anti-neurofilament medium polypeptide (anti-NFM, 1:1500, GTX133215; both from GeneTex, Irvine, CA, USA), rabbit polyclonal anti-Ephrin type B receptor (EPHB4, 1:1000, 20,883–1-AP; Proteintech, Rosemont, IL, USA). A mouse monoclonal antibody against GAPDH (GeneTex, 1:4000, GTX627408) was used to assess GAPDH expression as a loading control.

### General histology and immunohistochemical analysis

To prepare brain tissue for immunohistochemical evaluation, rats were anesthetized with 150 mg/kg ketamine and 30 mg/kg xylazine and perfused with cold 4% PFA in PBS. Brains were dissected, post-fixed for 48 h in 4% PFA and stored in sterile PBS at 4 °C. Coronal sections were prepared at 50 μm thickness with a VT1000S Vibratome (Leica Biosystems, Buffalo Grove, IL, USA). General brain histology was analyzed in hematoxylin/eosin (H&E)-stained sections.

For immunohistochemical analysis, sections were blocked with 10% normal goat serum in 50 mM Tris HCl, pH 7.6, 0.15 M NaCl, 0.3% Triton-X-100 and incubated overnight with the primary antibodies diluted in blocking solution at room temperature. After washing with PBS (6 times for 10 min each), sections were incubated with the appropriate Alexa (488 and 568)-conjugated secondary antibodies (1:300, ThermoFisher) in blocking solution for 2 h. After washing with PBS (6 times for 10 min each), the sections were mounted with Fluorogel mounting medium (Electron Microscopy Sciences, Hatfield, PA, USA). To visualize nuclei, sections were incubated in PBS containing 0.1 μg/ml DAPI (4′,6-diamidino-2′-phenylindole dihydrochloride) in the next to last PBS wash. The primary antibodies were a rat monoclonal anti-GFAP (1:500; gift of Virginia Lee, University of Pennsylvania, Philadelphia PA, USA), rabbit anti-GFAP (1:500, G9269), rabbit anti-NFH(1:500, N4142), rabbit anti-laminin (1:300, L9393), mouse anti-α-smooth muscle actin (α-SMA, 1:300, A2547) (all from Sigma-Aldrich), and mouse anti-phosphorylated NFH, (SMI31, 1:300, SMI 31P, BioLegend, San Diego, CA, USA). Primary negative controls consisted of omission of the primary antibodies. Images were collected with a Zeiss 700 confocal microscope (Jena, Germany) and processed with Adobe Photoshop CC (Adobe Systems, San Jose, CA, USA). Staining of the vasculature was performed with anti-collagen type IV antibodies (rabbit anti-rat collagen type IV, 1:300, ab6586, Abcam, Cambridge, MA, USA) as described elsewhere [30, 34]. Briefly, sections were incubated with 1 mg/ml pepsin (Agilent Technologies, Santa Clara, CA, USA) in 3% acetic acid for 50 min at 37 °C, washed with PBS, blocked and stained with antibodies against collagen IV and GFAP in combination with the respective Alexa-conjugated secondary antibodies as described above. For immunostaining of purified brain vascular preparations, freshly resuspended vascular fractions were fixed with 4% PFA in PBS overnight, rinsed with PBS, blocked as above and incubated overnight with biotin-labeled *Griffonia simplicifolia* isolectin B4 (0.3 μg/ml, L3759, Sigma-Aldrich). After washing with PBS, Alexa-568-conjugated streptavidin (1:300, Thermo Fisher) was added and incubated for 2 h at room temperature. After washing with PBS, vessels were transferred onto a slide, mounted with FluoroGel mounting medium and imaged with a Zeiss 700 confocal microscope.

### Electron microscopy

Electron microscopy (EM) was performed using protocols optimized to study the ultrastructure of the vasculature as previously described [14, 41, 44, 88]. Anesthetized rats were perfused as described above with cold 2% PFA containing 2% glutaraldehyde in 0.1 M sodium phosphate buffer, pH 7.0. Tissue was removed and post-fixed in the same fixative overnight. Fixed brains were placed on a rat brain slicer matrix, and coronal slices containing the frontal cortex were excised and processed. Slices were cryoprotected by immersion in 4% D-glucose, followed by increasing concentrations of glycerol (from 10 to 30% in phosphate buffer; *v*/v) and plunged rapidly into liquid propane cooled with liquid nitrogen (− 190 °C) in a Universal Cryofixation System KF80 (Reichert-Jung, Vienna, Austria). The samples were immersed in 0.5% uranyl acetate in anhydrous methanol (− 90 °C, 24 h) in a cryosubstitution AFS unit (Leica, Vienna, Austria). The temperature was raised from − 90 °C to − 45 °C in steps of 4 °C/h. After washing with anhydrous methanol, the samples were infiltrated with Lowicryl HM20 resin (Electron Microscopy Sciences, Fort Washington, PA, USA) at − 45 °C. Polymerization with ultraviolet light (360 nm) was performed for 48 h at − 45 °C and then for 24 h at 0 °C. Ultrathin sections (70 nm) were cut with a diamond knife on a Reichert-Jung ultramicrotome and mounted on nickel grids using a Coat-Quick adhesive pen (Electron Microscopy Sciences). Sections were imaged on a Hitachi 7700 electron microscope (Tokyo, Japan) and photographed with an Advantage CCD camera (Advanced Microscopy Techniques, Danvers, MA, USA). Image brightness and contrast were adjusted using the Adobe Photoshop CC software. Images were analyzed by an investigator blinded to the condition of the animal.

### High-resolution micro-computed tomography (micro-CT) scanning

Anesthetized rats were transcardially perfused with 250 ml of a 30% solution of the Brite Vu contrast agent (Scarlet Imaging, Murray UT, USA) maintained at 65–70 °C. The perfused animals were chilled by immersion in an ice-water bath for 60 min to gel the intravascular contrast agent. Brains were then dissected and post-fixed overnight in 4% PFA in PBS. Brains were scanned at a 7.5-μm voxel size using 50 kVp and 200 μA X-ray settings with exposure time of 810 ms per frame, with 3 frames averaged at each projection angle with a Bruker SkyScan 1272 micro-CT (Microphotonics, Allentown, PA, USA). 3D reconstruction was performed with Bruker’s CTVox 3D visualization software.

### Quantification of the cerebral vasculature

Morphological profiling of the cerebral vasculature was performed with the Vesselucida 360 software (v2018.1.1, MBF Bioscience, Williston, VT, USA) using data obtained from the micro-CT scans and automatically reconstructing the respective 3D vascular networks. Automatic reconstruction of the vasculature was performed using identical settings for all animals using the voxel scooping algorithm with the following settings: trace and seed sensitivity set to 80, medium seed density with refine filter set to 2, maximum gap tolerance. No manual editing was performed. The vascular parameters determined were total length, total surface area and total volume. Analysis was conducted by an investigator blinded to the condition of the animal.

### Statistical analyses

Statistical differences were evaluated with unpaired *t*-tests using Prism 7.0 software (GraphPad, La Jolla, CA). Statistical significance was set at an α level of 0.05.

## Results

### A rat model of low-level blast injury

We have been studying an animal model developed to mimic a level of blast exposure that would be associated with human mild TBI or a subclinical exposure. Initial studies using this system established that exposures up to 74.5 kPa (equivalent to 10.8 psi), caused no post-exposure apnea or mortality [2]. While representing a level of blast that is transmitted to the brain [2, 15], these exposures produced only mild transient behavioral disturbances and no obvious general brain histopathology. Histological examination of the lung showed no hemorrhages nor other pathology [2]. By contrast higher exposures (17.4 psi) led to frank subdural and intraparenchymal hemorrhages and visibly evident histopathology in brain along with pulmonary hemorrhages [2].

**Fig. 1:**
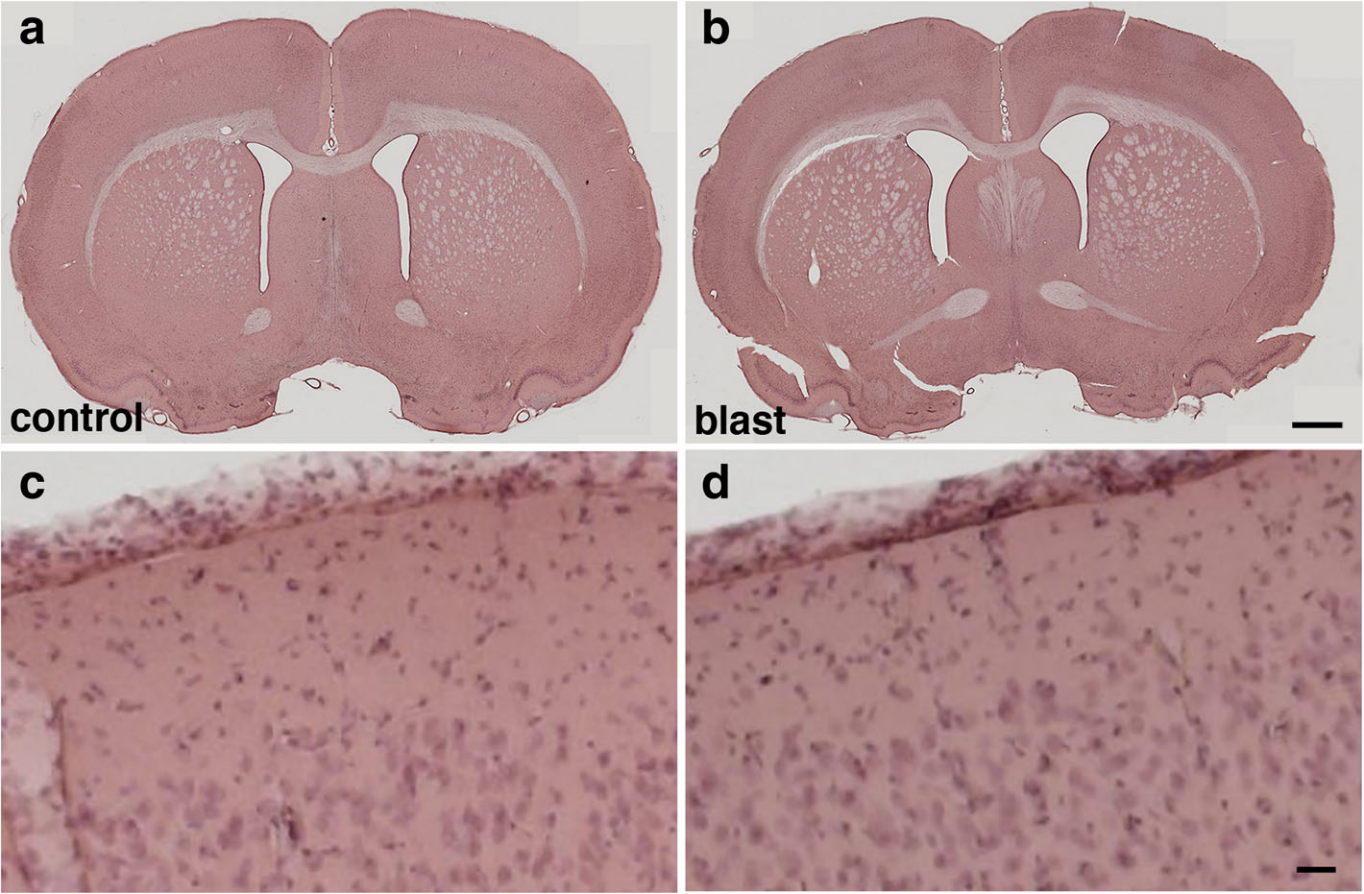
Lack of overt histopathology in the brain at 6 weeks after the last blast exposure. **a-d** Sections from control (**a**, **c**) and blast-exposed (**b**, **d**) animals were stained with hematoxylin and eosin. Rats were euthanized 6 weeks after blast exposure. Higher power images of the frontal motor cortex are shown panels (**c)** and (**d)**. Scale bars, 500 μm (**a**, **b**); 40 μm (**c**, **d**)

Because multiple blast exposures have been common among veterans returning from Iraq and Afghanistan [28], for most studies we used a design in which rats received three 74.5-kPa exposures delivered one exposure per day on 3 consecutive days. In multiple studies using this design we confirmed the lack of neuronal pathology at the light and EM levels as well as the absence of any reactive astrocytosis or general inflammatory reaction [2, 22, 26, 31–33] despite the fact that these animals develop chronic PTSD-related behavioral traits [26, 64–67]. Thus based on our experience presented in a number of published studies we believe that these blast pressures mimic a low-level blast exposure equivalent to a human mTBI or subclinical blast exposure. Examples of H&E stained sections from rats studied 6 weeks after blast exposure are shown in Fig. 1.

### Reduced GFAP expression and fewer astroglial attachments in isolated vascular fractions from blast-exposed rats at 6 weeks after blast exposure

**Fig. 2:**
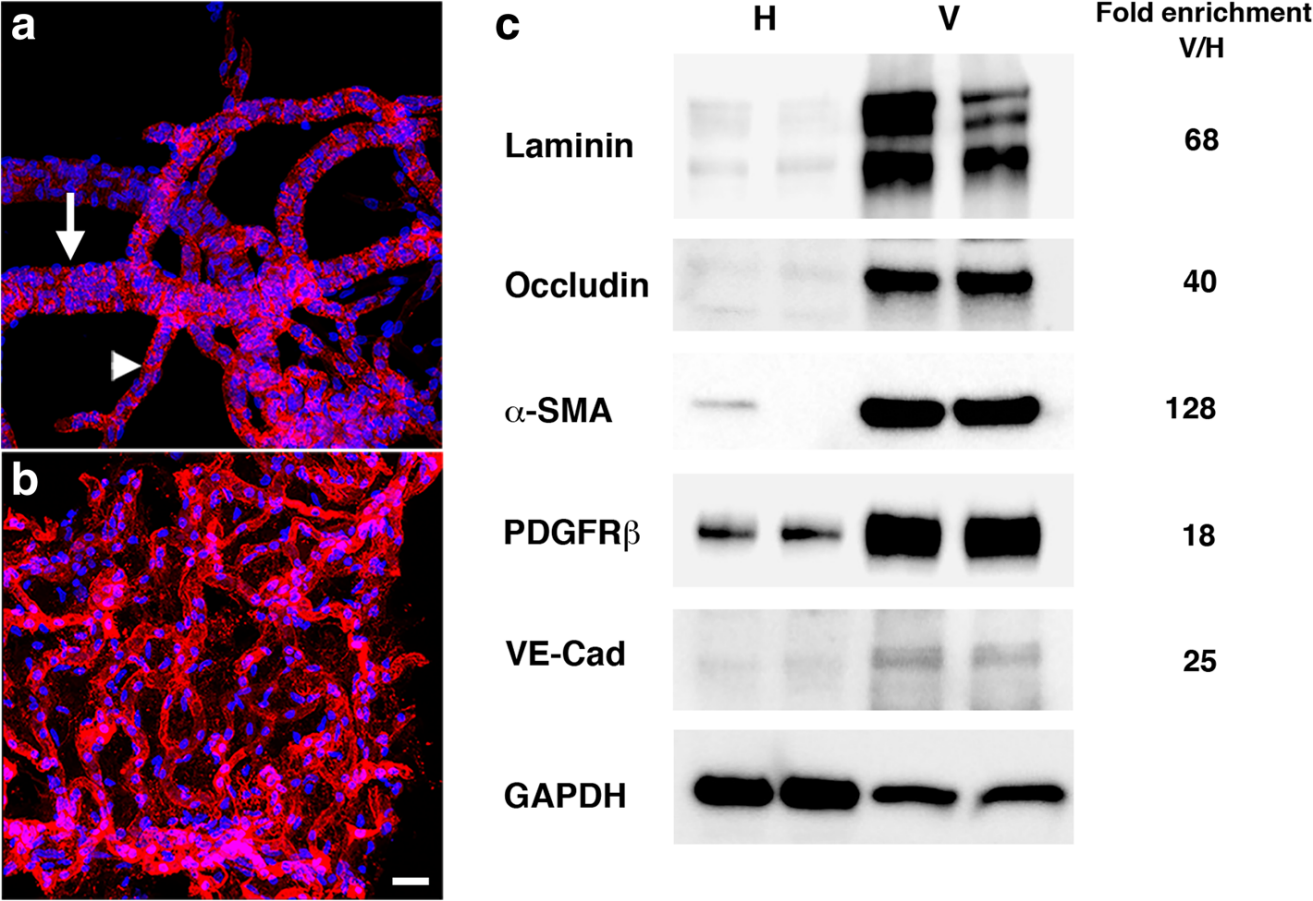
Immunohistochemical and biochemical characterization of purified vascular fractions from rat brain. **a-b** Isolated brain vascular fractions from a 3 month-old non-blast exposed rat is shown stained with *Griffonia simplicifolia* isolectin B4 (red). Nuclei were counterstained with DAPI (blue). **a** Muscular artery (indicated by an arrow) gives rise to a medium-sized vessel (indicated by an arrowhead). **b** Medium-and small-sized vessels and microvessels. Scale bar, 50 μm. **c** Immunoblots of total brain homogenates and vascular-enriched preparations are shown blotted with antibodies against endothelial (laminin, occludin, VE-cadherin), vascular smooth muscle (α-SMA), and pericyte (PDGFRβ) markers. All lanes were loaded with 10 μg of protein. Fold-enrichments in the vascular preparations vs. the whole brain homogenates are indicated on the right. Note enrichment of all the markers tested in the brain vascular fractions

Despite the generally benign appearance of the brain seen at the level of standard histopathology, EM analyses revealed that the brain vasculature in this model is particularly sensitive to blast injury [31]. To explore molecular changes in the vasculature of blast-exposed rats, we devised a method to isolate enriched vascular fractions from rat brain. Examples of isolated brain vascular fractions from non-blast exposed  adult Long-Evans rats are shown in Fig. 2. Microscopically enriched preparations contained a mixture of variously sized vessels linked in networks (vascular trees) that included arteries, arterioles, veins, venules and capillaries (Fig. 2a-b). Western blotting with antibodies against the vascular extracellular matrix protein laminin showed an average 68-fold enrichment (in two independent preparations) relative to the initial total brain homogenates (Fig. 2c). A similar enrichment of other vascular related components was demonstrated, including endothelial (occludin, 40-fold; VE-cadherin, 25-fold), pericyte (platelet-derived growth factor receptor β [PDGFRβ], 18-fold), and vascular smooth muscle (α-smooth muscle actin [α-SMA], 128-fold) markers (Fig. 2c).

**Fig. 3:**
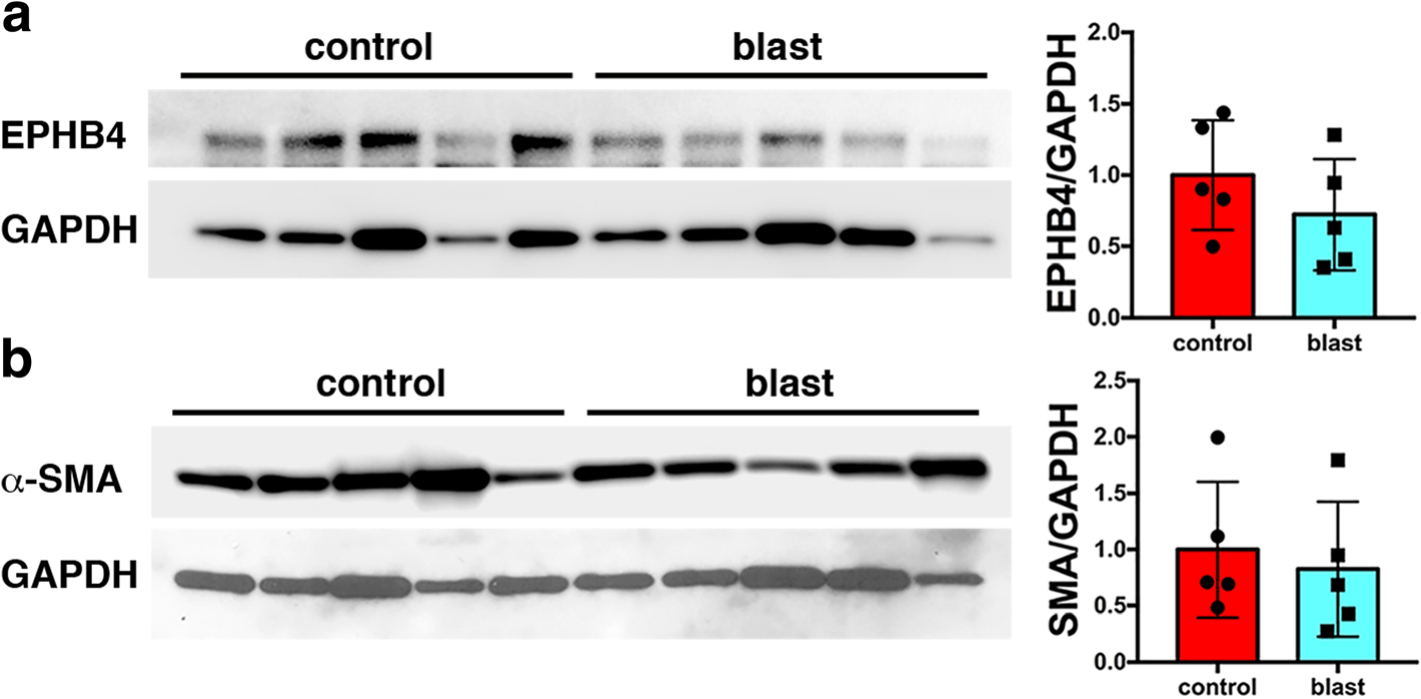
Comparison of expression of venous and arterial markers in vessels isolated from control and blast-exposed rat brains 6 weeks after the final blast exposure. **a-b** Western blotting showing expression of EPHB4 (**a**) and α-SMA (**b**) in isolated vascular fractions from five control and five blast-exposed animals. GAPDH was used as a loading control. All lanes were loaded with 10 μg of protein and contain protein from separate animals. Quantitation of the blots is shown in the right panels with expression normalized to GAPDH. There were no statistically significant differences (unpaired *t*-tests, *n* = 5/group)

Proteomic studies were conducted using purified brain vascular fractions isolated from control and blast-exposed rat brains at 6 weeks after the last of three consecutive daily blast exposures that were delivered when the animals were 10 weeks old. To determine the relative composition of the vascular fractions isolated from control and blast-exposed brains, we analyzed them by Western blotting with antibodies against α-SMA (a preferential marker of arterial smooth muscle) and EPHB4 (a marker of venous endothelial cells) [24]. No differences were noted between vascular preparations from blast-exposed and control rats (Fig. 3) suggesting that the relative composition of arterial and venous elements isolated is not affected by blast exposure.

**Fig. 4:**
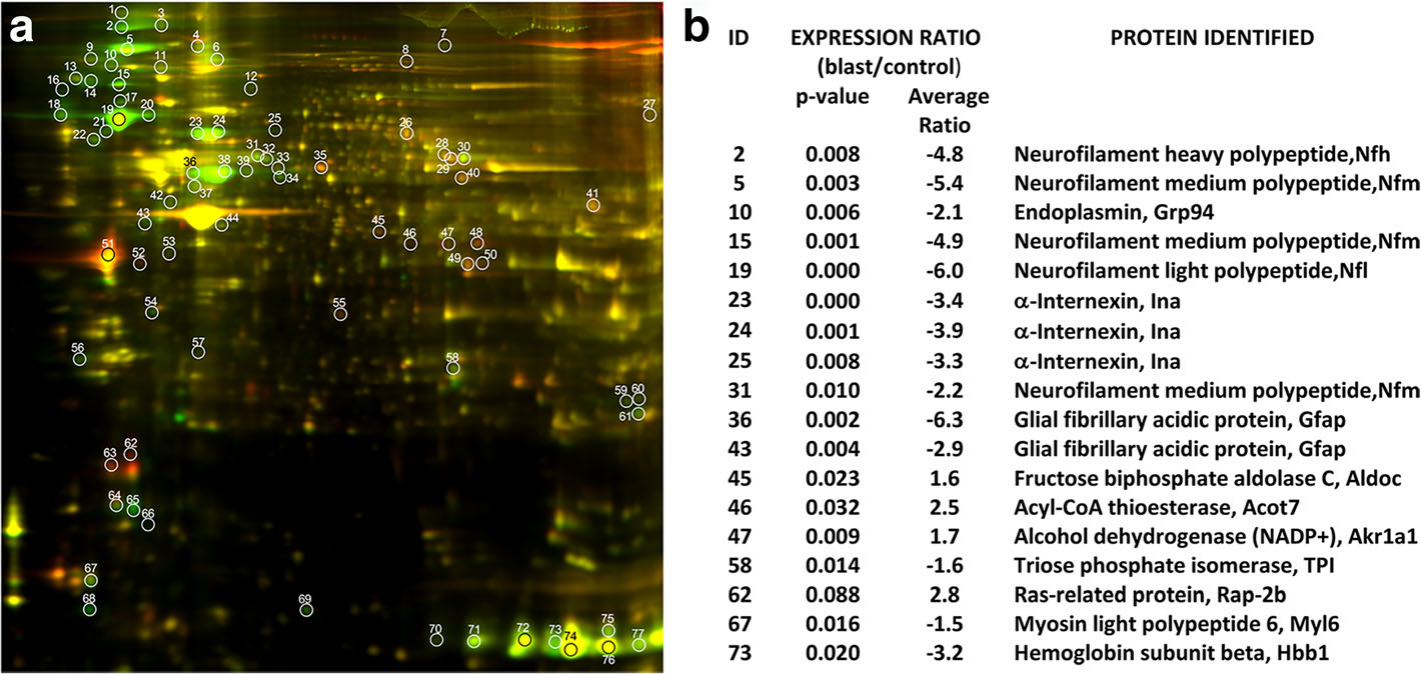
Expression profile of Cy-dye labeled proteins in vascular fractions prepared from brains of blast-exposed and control animals 6 weeks after blast exposure. **a** Representative 2D-DIGE image showing overlay of proteins from isolated vascular fractions of control (Cy-3 labeled, green spots) and blast-exposed (Cy-5 labeled, red spots) animals. White circles indicate differentially expressed proteins identified by image analysis with the DeCyder software. Each gel was loaded with 10 μg of protein. **b** Table showing the 18 spots identified by MS which represent a total of 13 distinct proteins. ID numbers correspond to the spot number in the gel image in panel (**a**). Average fold changes and *p*-values in blast relative to control are indicated

Protein profiles from two blast-exposed and two control animals were compared by 2D-DIGE (Fig. 4a). Using a cut-off level of 1.5, a total of 77 proteins showed altered expression in vascular preparations from the blast-exposed compared with controls. Based on fold-change between blast-exposed and control as well as calculated *p*-values, 18 proteins were selected for identification by MS. This analysis identified 13 distinct proteins (Fig. 4b).

**Fig. 5:**
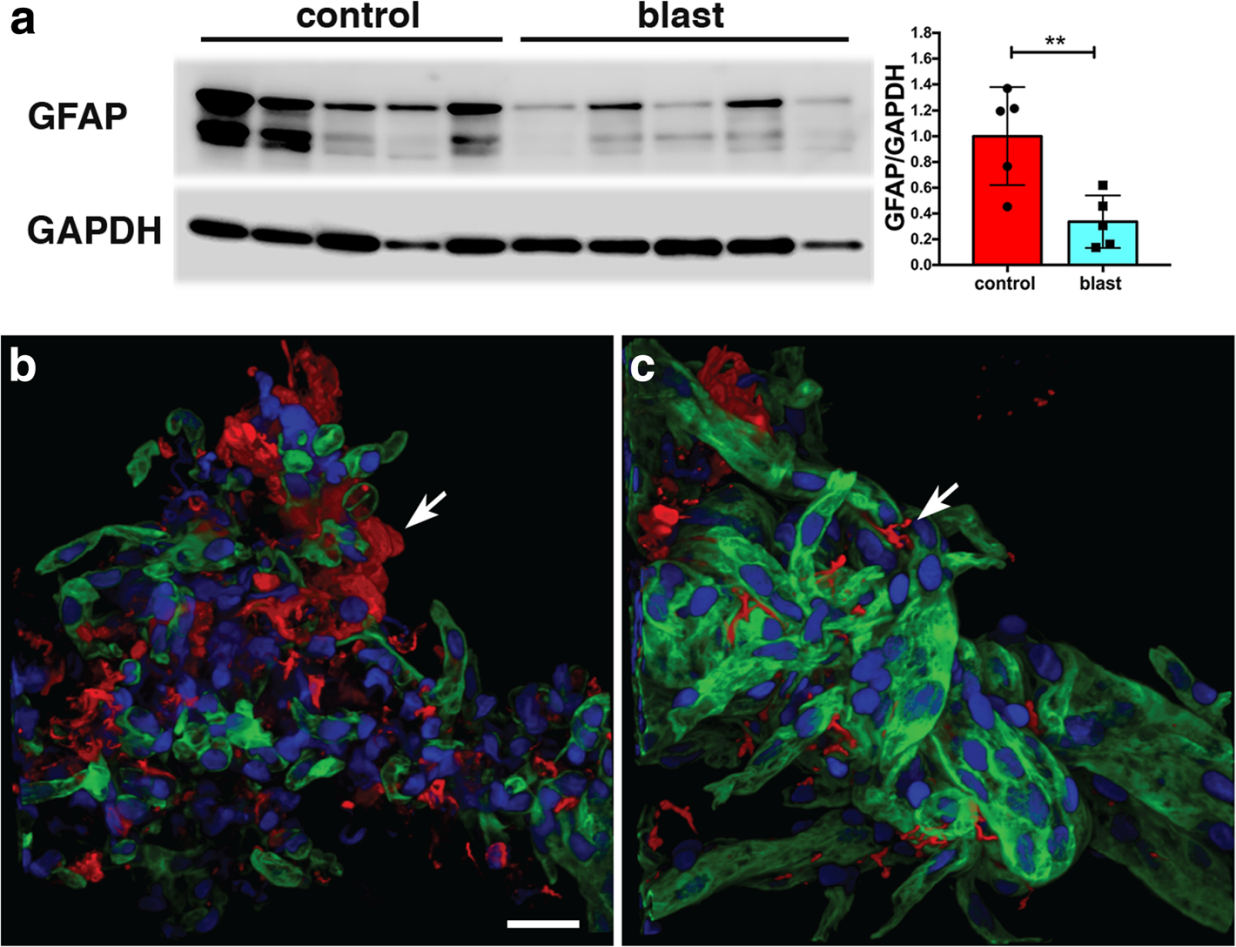
Reduced GFAP and fewer astroglial attachments in isolated brain vascular fractions following low-energy blast exposure. **a** Immunoblotting for GFAP (top panel) from five control and five blast-derived brain vascular fractions. Quantification of the blot with expression normalized to GAPDH (lower panel) is shown on the right. All lanes were loaded with 10 μg of protein and contain protein from individual animals. ** indicates *p* < 0.01 (unpaired *t*-test). **b-c** Isolated vascular fractions from control (**b**) and blast-exposed brains (**c**) were immunostained for GFAP (red) and laminin (green) and counterstained with DAPI (blue). Arrows indicate GFAP-immunostained attachments to the isolated vasculature. Note the reduced number of GFAP-labeled attachments in the blast-exposed vessels (**c**). All animals were euthanized 6 weeks after blast exposure. Scale bar, 25 μm

Surprisingly, the protein showing the most altered expression was GFAP, which was decreased in blast-exposed animals. To confirm the proteomic data, levels of GFAP in isolated vessels from control and blast-exposed animals were determined by Western blotting. As shown in Fig. 5a, Western blotting confirmed that GFAP expression was decreased (about 3-fold) in the isolated brain vascular fractions from blast-exposed rats.

**Fig. 6:**
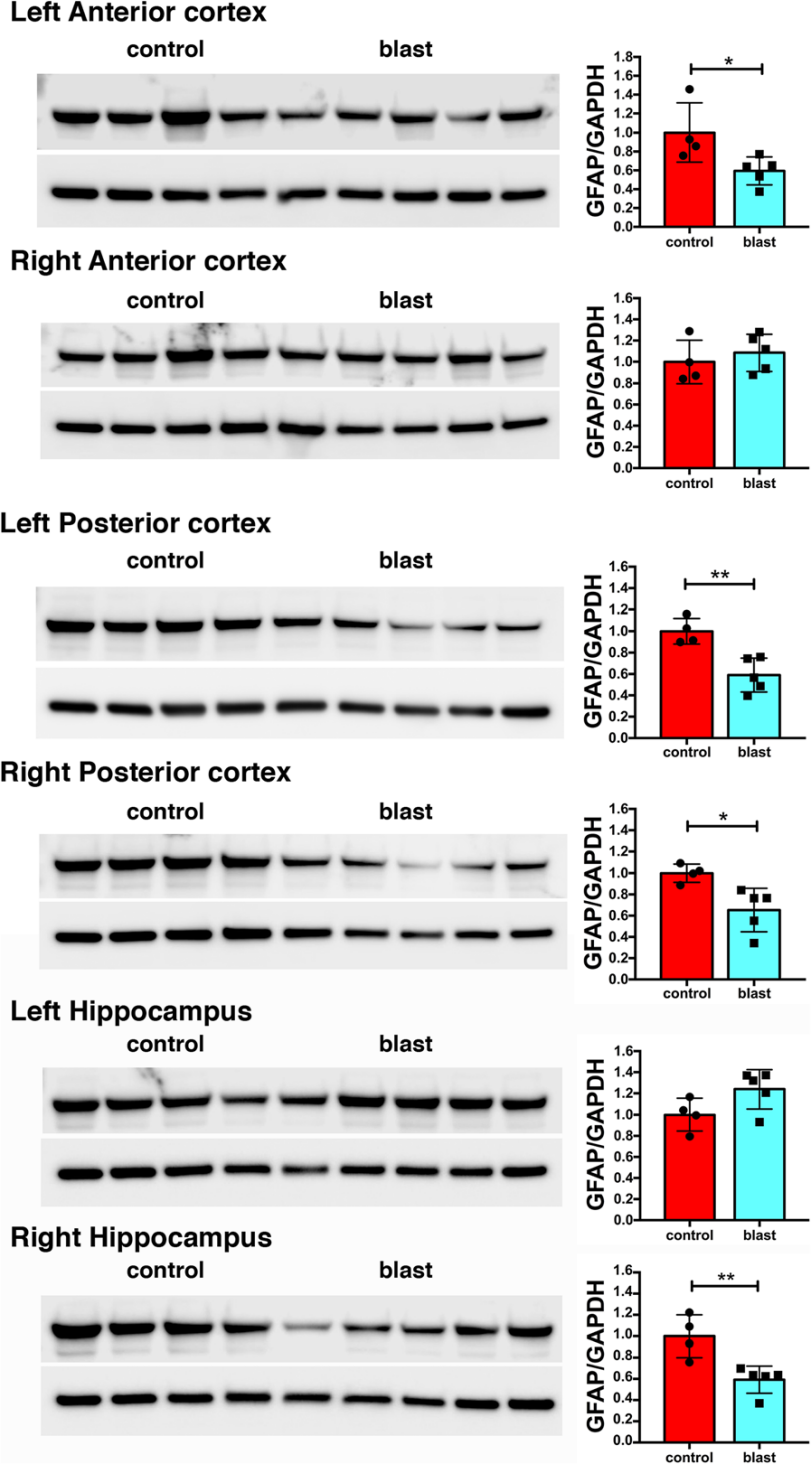
GFAP levels in whole brain extracts following low-energy blast exposure. Immunoblotting for GFAP in extracts of brain regions from four control and five blast-exposed rats. Levels of expression were normalized to GAPDH as shown in the bar graphs. All lanes were loaded with 50 μg of protein and represent individual animals. Statistical differences were assessed with unpaired *t*-tests (* *p* < 0.05, ** *p* < 0.01). All animals were euthanized 6 weeks after blast exposure

The presence of GFAP in isolated vascular fractions suggested that astroglial endfeet were remaining attached to vessels, with the reduction of GFAP in blast-exposed samples reflecting loss of these attachments. To determine whether astroglial attachments could be visualized in isolated vascular fractions, we performed GFAP immunostaining (Fig. 5b-c). In controls (Fig. 5b), GFAP staining occurred in patches, consistent with perivascular attachment of astroglial endfeet. Others have also observed astrocytic endfeet attached to purified mouse vasculature [10, 20, 77]. In blast-exposed rats (Fig. 5c), perivascular GFAP staining also occurred in patches but was noticeably reduced compared to controls, consistent with astroglial endfeet being lost. To determine whether changes in GFAP levels could be detected in brain tissue we performed Western blotting on selected subregions (anterior cortex, posterior cortex, and hippocampus) harvested 6 weeks after blast exposure. As shown in Fig. 6, GFAP was decreased in the left anterior cortex as well as the left and right posterior cortex and the right hippocampus (about 1.7 fold).

### Reduced neuronal intermediate filament protein levels in vascular preparations from blast-exposed rats at 6 weeks following blast exposure

**Fig. 7:**
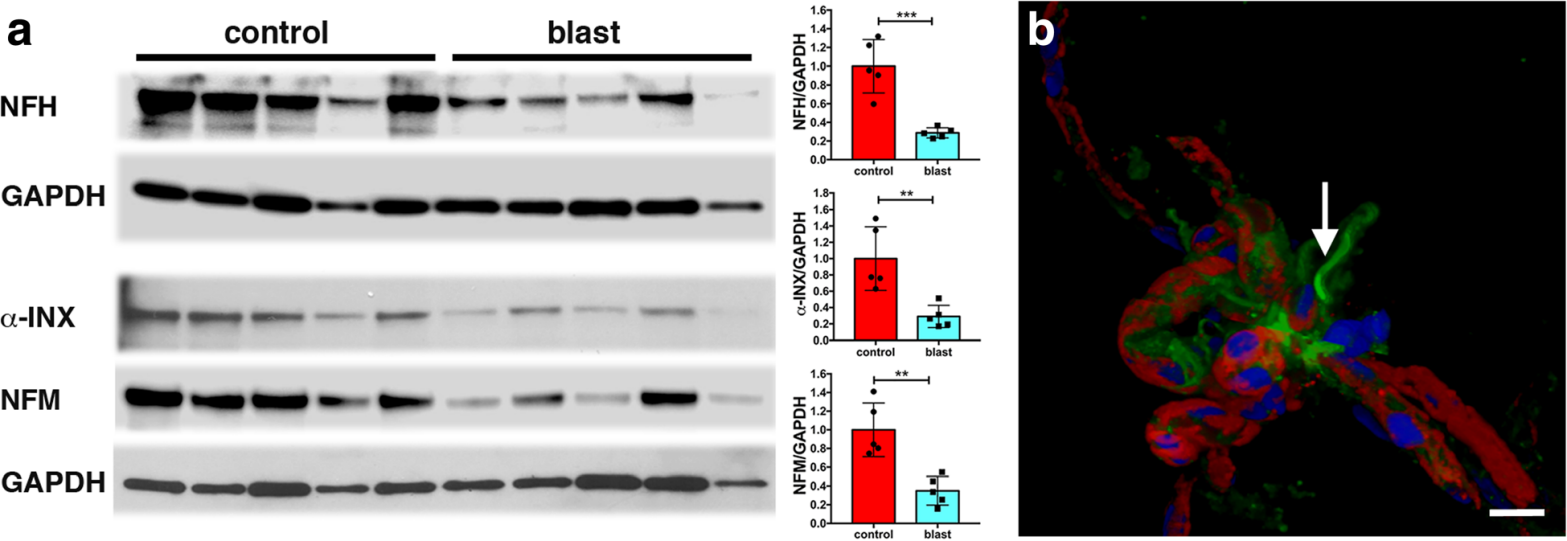
Disruption of neurovascular interactions by low-energy blast exposures. Isolated brain vascular fractions were prepared from five control and five blast-exposed rats (6 weeks after blast exposure, same samples as shown in Fig. 5a). **a** Western blot analysis of the expression of NFH, α-INX and NFM in isolated vascular fractions. The top GAPDH panel represents the loading control for the NFH blot; the bottom GAPDH panel is the loading control for the α-INX and NFM blots. Blots were obtained by sequential probing of the same membrane. All lanes were loaded with 10 μg of protein and contain protein from individual animals. Quantitation is shown in the right panels with expression normalized to GAPDH. Statistical differences were assessed with unpaired *t*-tests (** *p* < 0.01, *** *p* < 0.001, *n* = 5/group). **b** Isolated large cerebral vessel, likely a pial or penetrating arteriole, stained for NFH (green) and *Griffonia simplicifolia* isolectin B4 (red). Nuclei were stained with DAPI (blue). An arrow indicates an NFH-immunostained process that remains attached to the vessel. Other patches of focal NFH immunoreactivity are also visible. Scale bar, 20 μm

Somewhat surprisingly, besides GFAP, 4 of the first 9 proteins identified by MS were intermediate filament proteins (IFs) (Fig. 4b), but rather than being glial or endothelial in origin they were neuronal-associated IFs (α-INX and the light, medium and heavy polypeptide neurofilaments). All were decreased in blast-exposed vessels based on 2D-DIGE. Figure 7a shows Western blot analysis of the isolated vascular fractions (the same samples shown in Fig. 5a) with antibodies against NFH, α-INX, and NFM. In each case as in the proteomic studies, the levels of neuronal IFs were decreased by about 3-fold in blast-exposed rats.

The presence of neuronal IFs in isolated vascular fractions suggested that neuronal fibers remain attached or at least tightly associated with purified brain vascular preparations. Immunostaining of vascular fractions for NFH showed focal staining sometimes with fine processes apparent, consistent with neuronal attachments to blood vessels (Fig. 7b). Our results are in agreement with others who have also observed neuronal IFs in highly purified preparations of mouse brain vasculature [10, 20]. Their decrease following blast injury suggests that, like gliovascular, neurovascular attachments are being disrupted as a consequence of blast injury.

### Astroglial coverage of blood vessels in brain at 6 weeks after blast exposure

**Fig. 8:**
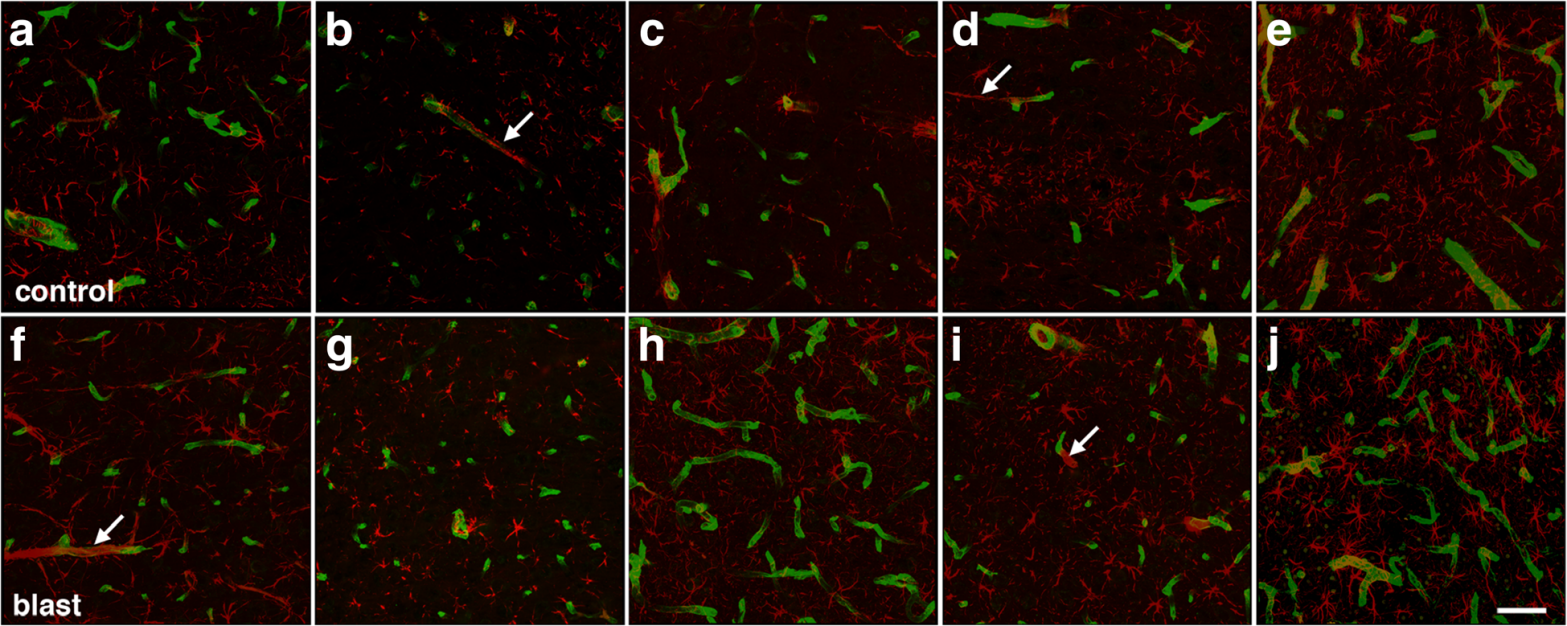
Visualization of astrocyte coverage of the brain vasculature in control and blast-exposed rats. Sections from five individual control and five blast-exposed animals were immunostained for collagen IV (green) and GFAP (red) utilizing an antigen retrieval protocol employing pepsin treatment [30, 34]. Rats were euthanized 6 weeks after blast exposure. **a**-**e** Representative images from the prelimbic cortex from each individual control brain. **f**-**j** Representative images from the prelimbic cortex from each individual blast exposed brain. Arrows in panels (**b**, **d**, **f**) and (**i**) indicate examples of perivascular astroglial fibers associated with domains on blood vessels that stained poorly with antibodies against collagen IV even after pepsin treatment. Scale bar, 40 μm

**Fig. 9:**
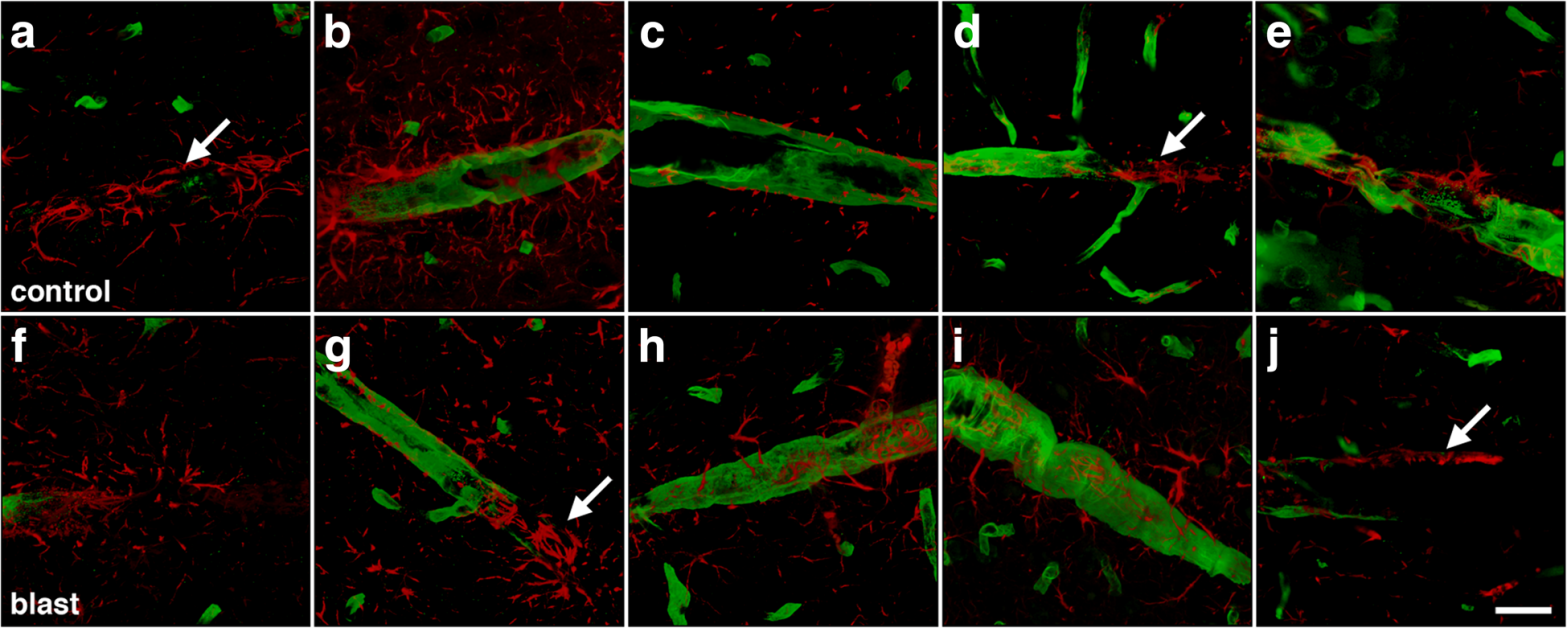
Astrocyte coverage of large and medium sized parenchymal vessels in control and blast-exposed animals euthanized 6 weeks after exposure. Brain sections of five control and five blast-exposed animals were immunostained for collagen IV (green) and GFAP (red). **a**-**e** Representative images in the prelimbic cortex from each individual control animal. **f**-**j** Representative images in the prelimbic cortex from each individual blast-exposed animal. Arrows in panels (**a**, **d**, **g**) and (**j)** indicate areas of perivascular GFAP expression in regions of blood vessels that stain poorly for collagen IV. Scale bar, 20 μm

The loss of gliovascular attachments in isolated vascular fractions would predict less astrocyte coverage of blood vessels in intact brain. To estimate astroglial coverage of blood vessels in brain, sections of control and blast-exposed animals were initially analyzed by GFAP and collagen IV immunohistochemical staining. Figures 8 and 9 show representative sections of the prelimbic cortex from five blast-exposed and five control animals euthanized at 6 weeks after blast exposure. No obvious differences were seen in any brain region examined between blast-exposed and control specimens.

While others have quantified astroglial coverage of human microvessels in brain by assessing the colocalization of GFAP and collagen IV immunostaining [6, 70], we found that this technique was not directly applicable to rat brain since vascular segments greatly enriched with GFAP immunoreactivity were very often present in areas devoid of collagen IV immunoreactivity (Figs. 8 and 9). This was observed in sections from both controls and blast-exposed rats, even when the tissue was subjected to a protease antigen retrieval protocol utilizing pepsin to unmask vascular collagen IV immunostaining [30, 34].

**Fig. 10:**
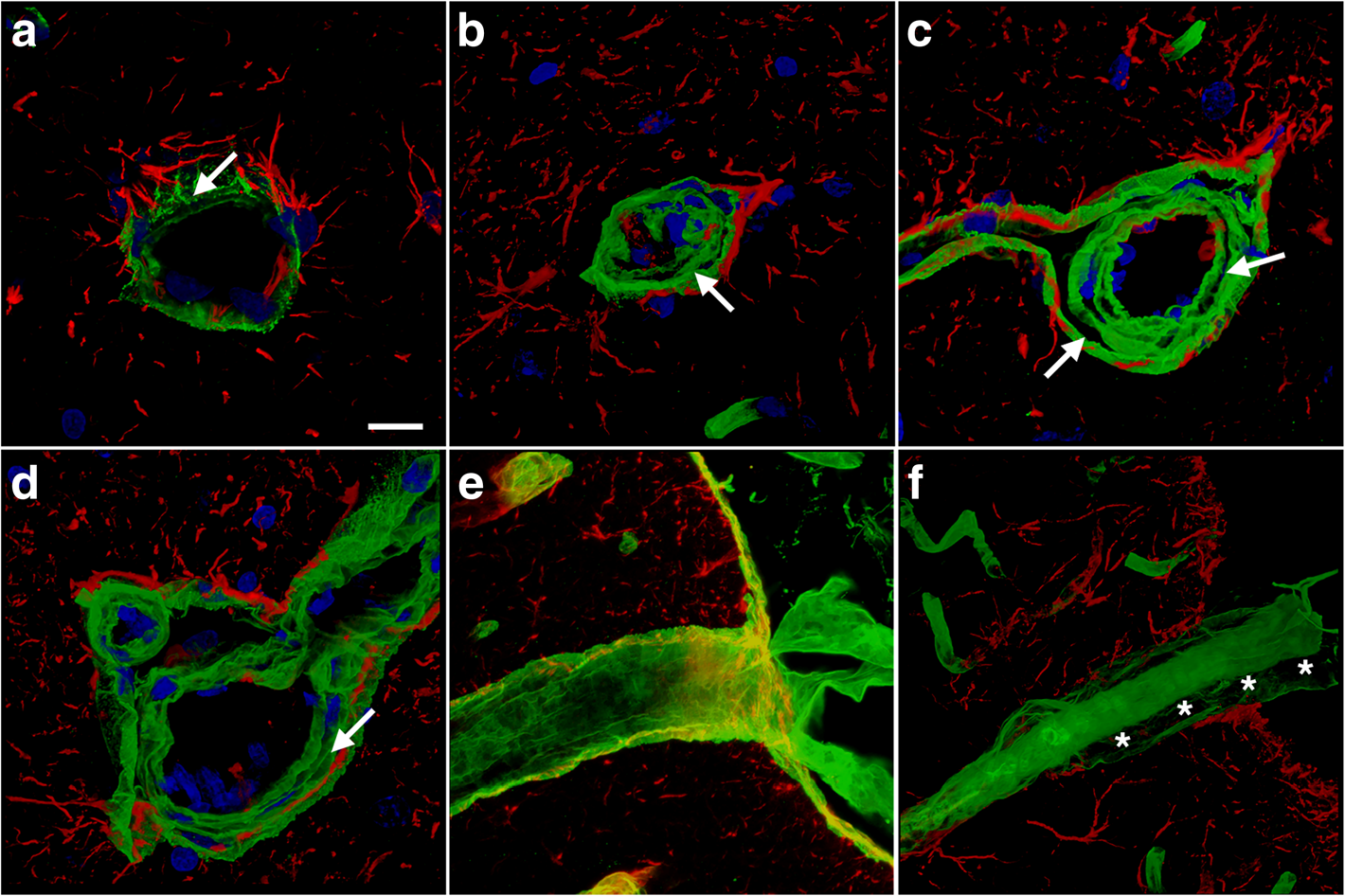
Altered vascular extracellular matrix in blast-exposed animals. Brain sections of rats euthanized 6 weeks after blast exposure were immunolabeled for collagen IV (green) and GFAP (red) with DAPI nuclear staining (blue). Arrows in the panels indicate the collagen IV-rich layers, which include the endothelial basal membrane and adventitia. **a**-**d** Representative sections from the hippocampal stratum lacunosum moleculare from a control (**a**) and a blast-exposed rat (**b**-**d**). Note the separation of the collagen IV-rich layers in panels (**b**-**d**), resulting in a multilayered appearance of the collagen IV- immunostained extracellular matrix. In panel (**b**) the loss of structure in the collagen IV-rich layers resulted in collapse of the lumen. **e**-**f** Penetrating cortical vessels from control (**e**) or blast-injured (**f**) rats. The blast-exposed vessel in panel (**f**) exhibits a double-barreled appearance. Asterisks (*) in panel (**f**) mark the separation of the adventitial layer from the tunica media of the blood vessel. Scale bar, 20 μm

Although it failed to provide direct evidence of altered gliovascular coverage in brain, collagen IV immunostaining frequently revealed other vascular alterations induced by blast exposure. Figure 10 shows examples of large penetrating vessels in the hippocampal lacunosum moleculare where collagen IV immunoreactivity revealed lumens that were irregular with a thickened and multilayered appearance, which were at times collapsed (Fig. 10b-d). Figure 10f also shows an example of a penetrating cortical vessel in which the external layers of the adventitia have separated giving the vessel a double-barreled appearance.

**Fig. 11:**
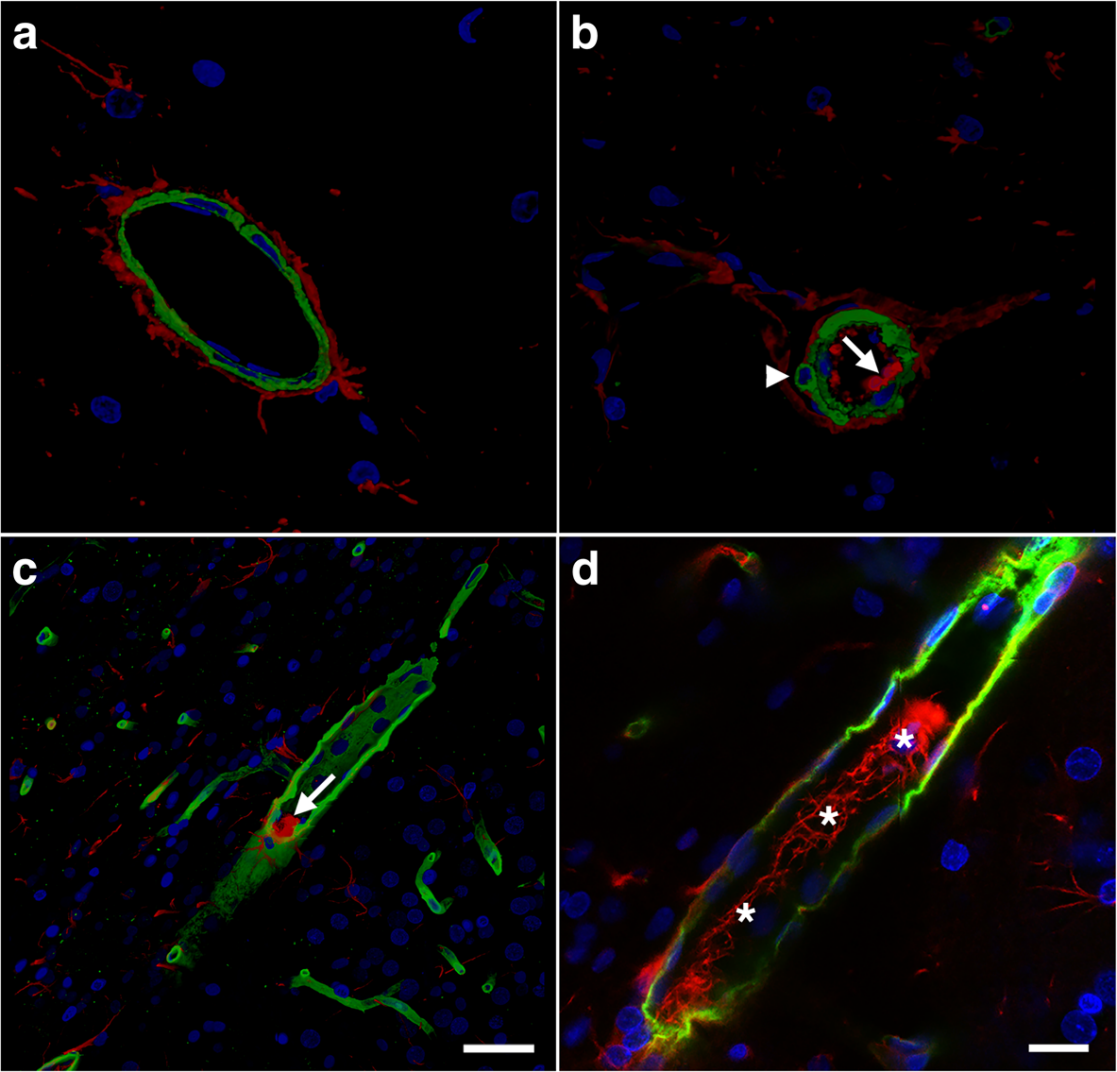
Intraluminal astrocytic processes after blast exposure. Brain sections from rats sacrificed 6 weeks post-blast exposure were immunostained for GFAP (red) and α-SMA (green) with a DAPI nuclear counterstain (blue). **a**-**d** Representative sections of the hippocampal stratum lacunosum moleculare from a control (**a**) or a blast-exposed (**b**) rat. The arrow in panel (**b**) indicates the presence of intraluminal GFAP. The arrowhead in panel (**b**) indicates vacuolation in the smooth muscle (α-SMA staining). The smooth muscle layer appears thick, irregular and disorganized compared to that in the control in panel (**a**). **c** Panoramic 3D reconstruction of a large parenchymal vessel in a blast-exposed animal exhibiting intraluminal GFAP expression (arrow). **d** A 0.56-μm-thick confocal optical section from the cell in panel (**c**) showing directionally oriented GFAP-immunostained processes (asterisks) within the lumen. Scale bars: 50 μm for (**a**-**c**), 20 μm for (**d)**

**Fig. 12:**
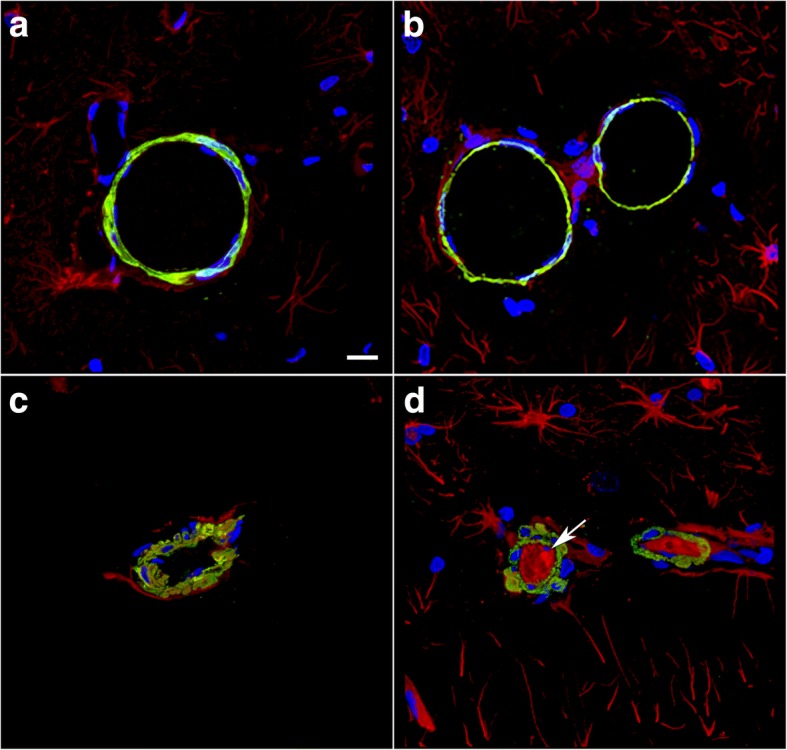
Disrupted smooth muscle layers and intraluminal astrocytic processes are present in vessels 10 months after blast. Brain sections from rats sacrificed 10 months after the last blast exposure were immunostained for GFAP (red) and α-SMA (green) with a DAPI nuclear counterstain (blue). **a**-**d** Representative sections of the hippocampal stratum lacunosum moleculare from control (**a**-**b**) or blast-exposed (**c**-**d**) rats. The arrow in (**d**) indicates the presence of intraluminal GFAP. Note the vacuolation in the smooth muscle (α-SMA staining). Scale bar, 50 μm

In addition, perivascular GFAP-containing processes often appeared disturbed. Figure 11 shows blood vessels in the lacunosum moleculare of the hippocampus immunostained for α-SMA and GFAP. Besides disturbed vascular smooth muscle (α-SMA), which often appeared irregular and vacuolated (Fig. 11b), intraluminal astrocytic processes were frequently visible (Fig. 11b-d). Figure 11d in particular shows a parenchymal vessel that exhibits a large nest of intraluminal GFAP-immunoreactive processes. Similarly, abnormal α-SMA immunostaining and intravascular GFAP-immunolabled processes were found in the brains of animals 10 months after blast exposure (Fig. 12).

### Degeneration of astrocytic endfeet in blast-injured brain at 6 weeks following blast exposure revealed by EM

**Fig. 13:**
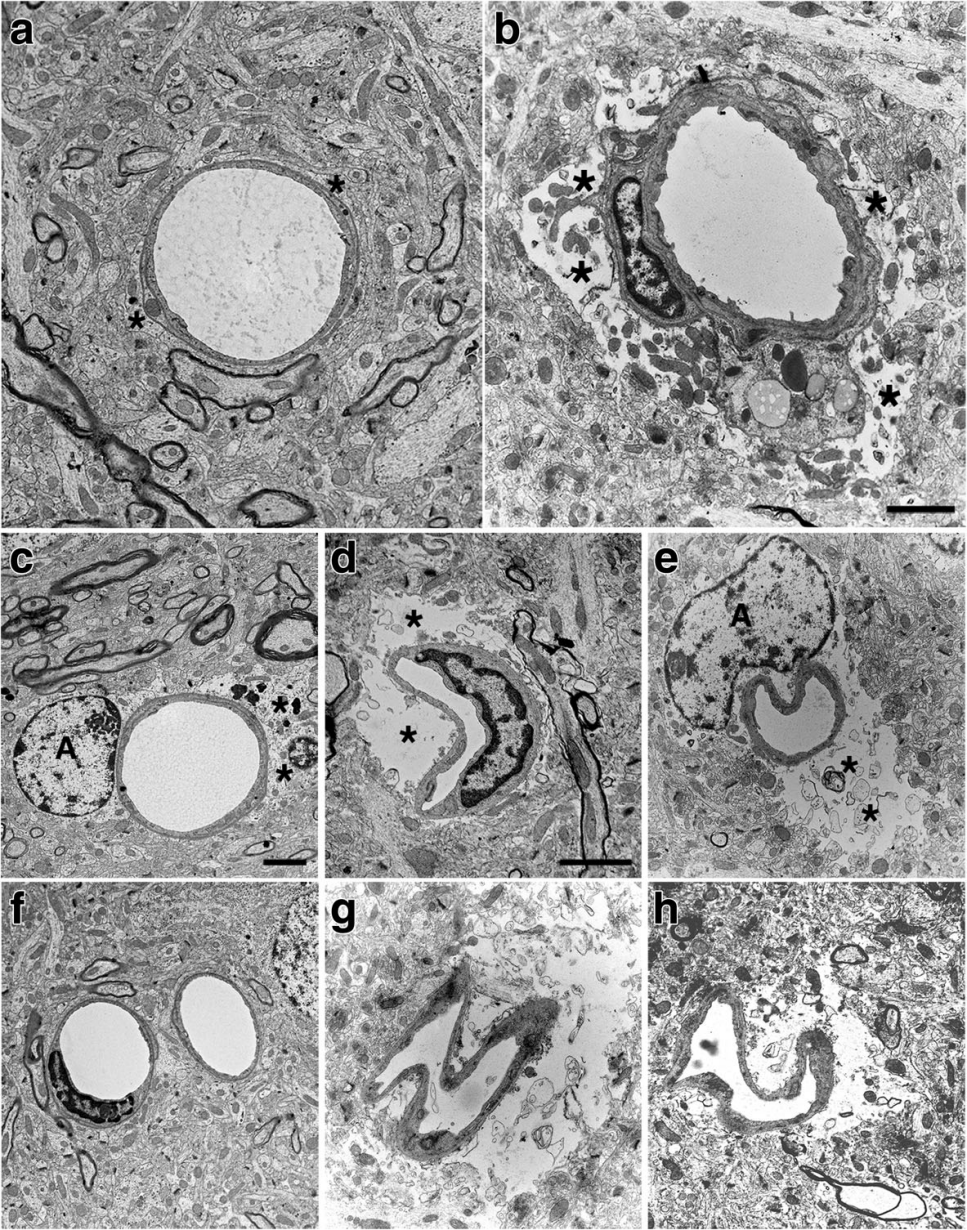
Blast induced swelling and degeneration of astrocytic endfeet in the frontal motor cortex. **a**-**f** Electron micrographs are shown from sections of the frontal motor cortex of control (**a**, **c**, **f**) and blast-injured (**b**, **d**, **e**, **g**, **h**) rats. Animals were euthanized 6 weeks following blast exposure. Asterisks (*) indicate astrocytic endfeet, which are swollen and contain degenerating organelles in all the blast-exposed microvessels. The lumens of the microvessels in the blast-exposed animals also appear irregular and collapsed. Perivascular astrocytes (A) are visible in panels (**c**) and (**e**). Scale bars, 2 μm. Scale bar in panel (**b**) applies to panels (**a**) and (**b**). Scale bar in panel (**c**) applies to panels (**c**, **e**, **f**, **g**) and (**h**). Scale bar in panel (**d**) applies only to panel (**d**)

To gain a better understanding of how blast affects gliovascular connections at the ultrastructural level we examined sections from the motor cortex of rats harvested at 6 weeks after the last blast exposure. Figure 13 shows examples of small arterioles from control and blast-exposed rats. Compared to the controls, the lumens of the blast-exposed vessels appeared irregular and thickened. In addition, compared to the tight astrocytic endfeet surrounding control vessels, perivascular astrocytic endfeet in the blast-exposed vessels were swollen and contained degenerating organelles. The lumens of the associated vessels were often irregular and collapsed. While the level of endfoot degeneration varied considerably, a count of 50 randomly sampled vessels in the blast-exposed specimen revealed that 9 (18%) had clearly swollen astrocytic endfeet such as those illustrated in Fig. 13. Blast-exposed capillaries showed similar changes. Astrocytic changes were more obvious in blood vessels showing the most endothelial cell damage. Such changes were not observed in microvessels of controls.

### Blast-induced vascular occlusion by CD34-immunoreactive cells in rats sacrificed 6 weeks after blast exposure

**Fig. 14:**
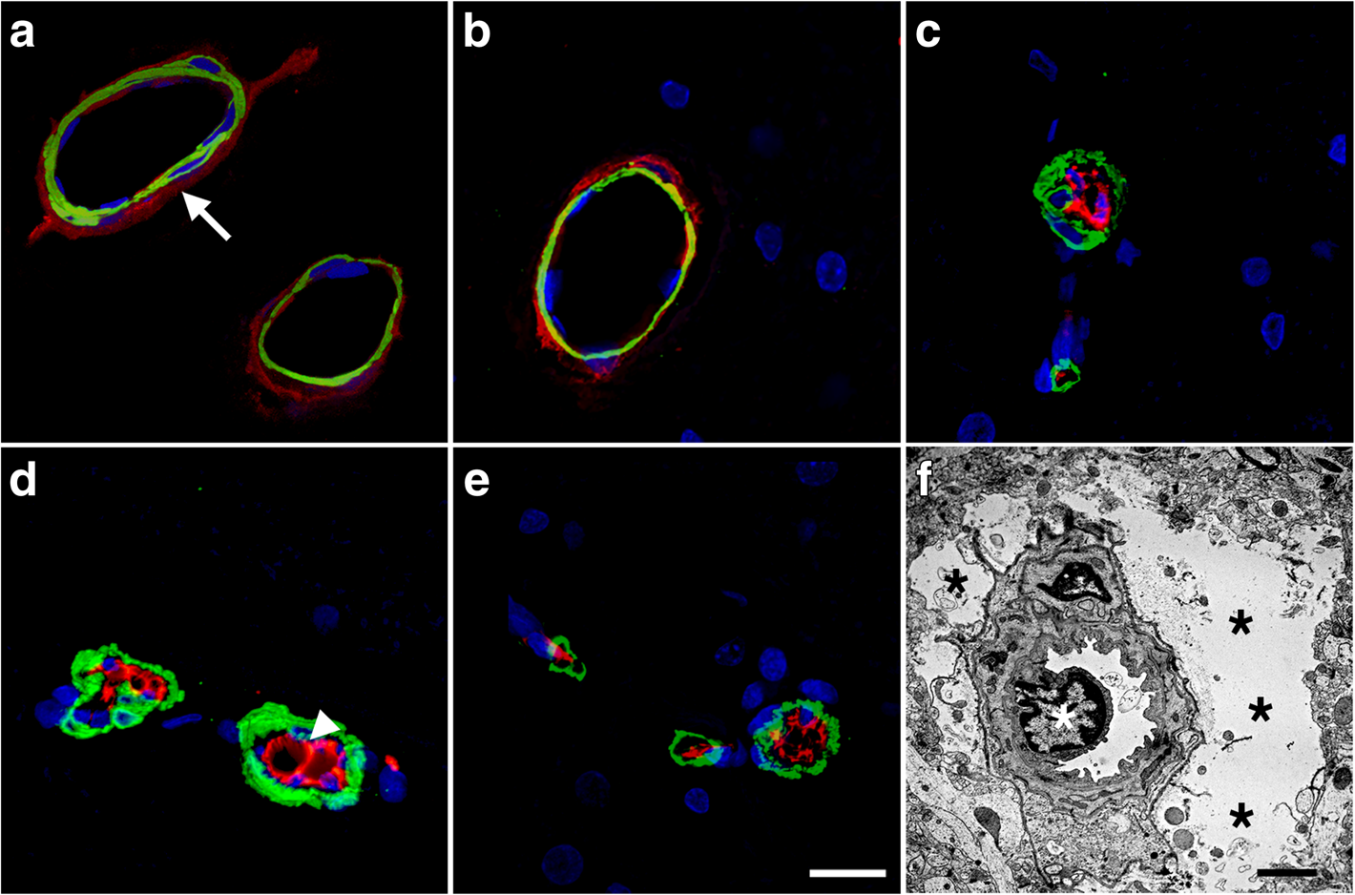
Vascular smooth muscle alterations and arteriolar occlusion by CD34-expressing cells. **a**-**e** Brain sections from control and blast exposed rats sacrificed 6 weeks after blast exposure were immunostained with antibodies against vascular α-SMA (green) and CD34 (red). Nuclei were counterstained with DAPI (blue). Representative sections of the hippocampal stratum lacunosum moleculare from control (**a**-**b**) and blast-exposed (**c**-**e**) rats. Note that CD34 immunoreactivity is present in the adventitia of control vessels (arrow in **a**) but is absent in the blast-injured vessels. The smooth muscle layer appears thickened and irregular in the blast-exposed vessels, and CD34-expressing cells are present inside the vessel lumen (arrow head in **d**). **f** Electron micrograph of a cortical arteriole from the frontal cortex of a blast-exposed rat showing a cellular occlusion (white asterisk) similar to those shown in panels (**c, d**) and **(e)**. Black asterisks (*) in panel (**f)** mark swollen astrocytic endfeet. Scale bars: 20 μm for (**a**-**e**), 2 μm for (**f**)

Some cerebral small vessels from blast-exposed animals also appeared to be occluded by CD34-expressing cells. Figure 14 shows sections double-immunostained for α-SMA and CD34. Unlike controls in which CD34 immunostaining was confined to the adventitia, in blast-exposed animals CD34-immunoreactive elements were seen occluding the vessel lumen. Occlusions could also be identified by EM in blast-exposed rats (Fig. 14f). As in Figs. 11 and 12, the α-SMA immunoreactivity in the blast-exposed microvessels shown in Fig. 14 appeared thickened, irregular and vacuolated, indicative of altered smooth muscle layers.

### Recovery of GFAP and neuronal IF expression in isolated brain vascular fractions from blast-exposed rats 8 months after exposure

**Fig. 15:**
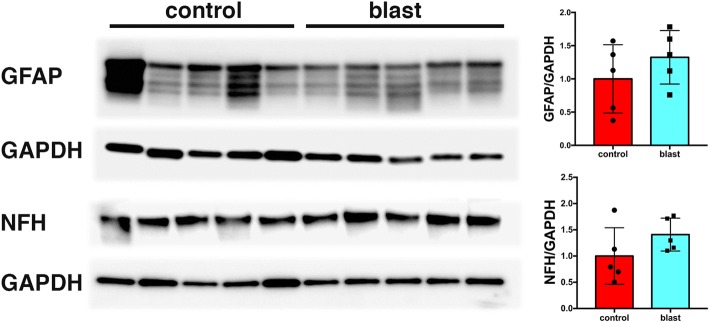
GFAP and NFH levels in blast injured vascular fractions recover with time following blast injury. Brain vascular fractions were isolated from five control and five blast-exposed rats 8 months after the last blast exposure. The figure shows immunoblotting for GFAP and NFH followed by reprobing for GAPDH. All lanes were loaded with 10 μg of protein and contain protein from individual animals. Quantification with expression normalized to GAPDH is shown on the right. No significant differences were observed between blast-exposed and control animals

To determine whether GFAP and neuronal IF expression remained chronically decreased in isolated brain vascular fractions following blast injury, we studied a group of blast-exposed and control rats 8 months following blast injury. As shown in Fig. 15, GFAP and NFH levels were unchanged in the blast-exposed compared to control samples.

### Chronic blast-induced cerebral vascular pathology at 10 months following blast exposure revealed by micro-CT

**Fig. 16:**
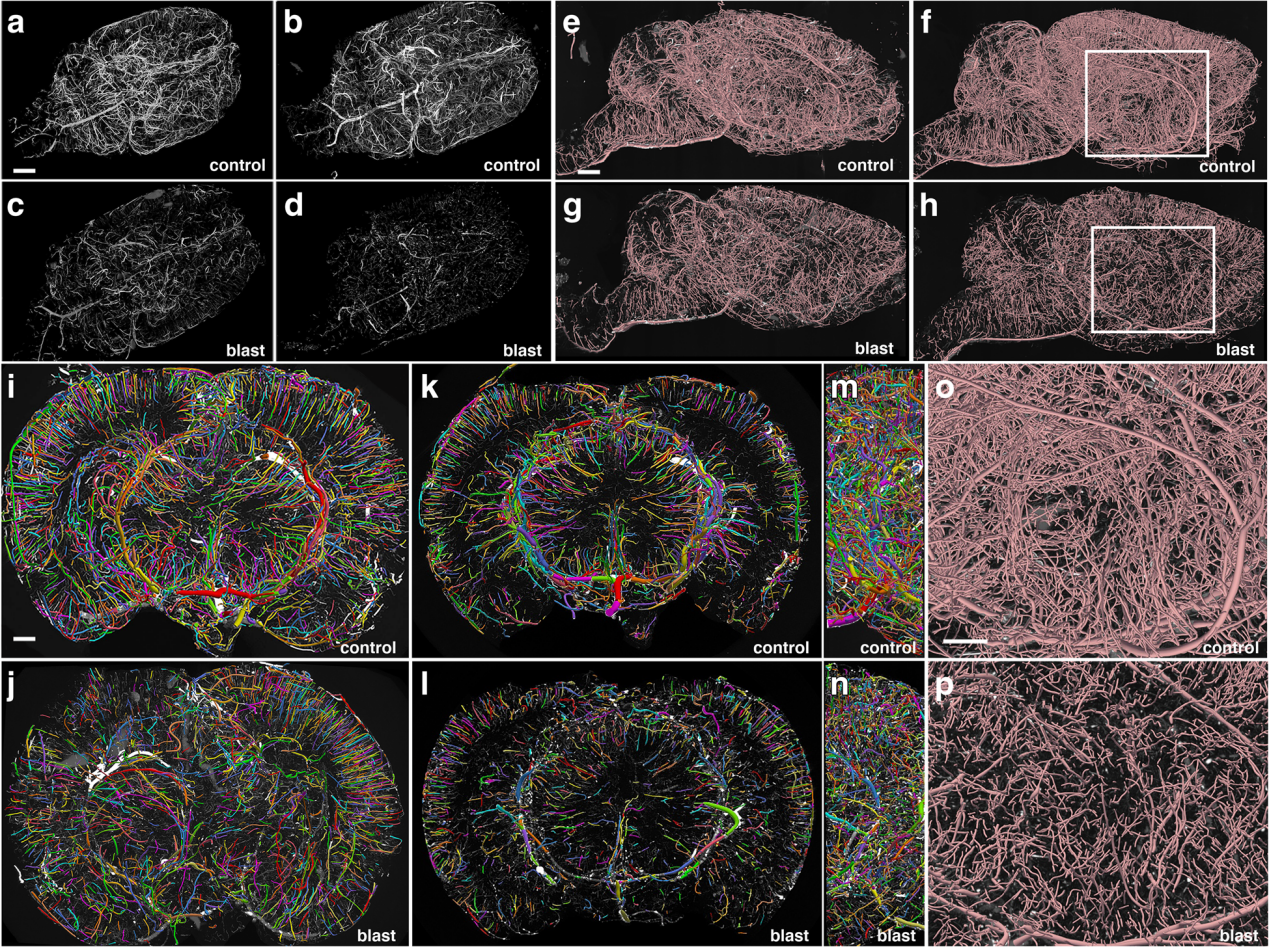
Chronic vascular pathology in blast-exposed rats revealed by micro-CT scanning. Two control and two blast-exposed rats were transcardially perfused with the Brite Vu contrast agent at 10 months after blast exposure. Brains were scanned at a resolution of 7.5 μm using equispaced angles of view around 360°, and 3D reconstructions were prepared with Bruker’s CTVox 3D visualization software. **a**-**d** MIP images of volume-rendered brain vasculature from two control (**a**, **b**) and two blast-exposed (**c**, **d**) rats revealed diffuse thinning of the brain vasculature in the blast-exposed rats. Scale bar, 2 mm. **e**-**h** Trace sagittal reconstructions used for the automated quantitation from control (**e**-**f**) and blast-exposed rats (**g**-**h**) **o**-**p** Higher magnification views of the regions outlined by the boxes in panels **(f)** and **(h)**. Scale bars, 1 mm for (**e**-**h**), and 0.6 mm for (**o**-**p**). **i**-**n** Reconstructions of coronal optical sections from the brains of control (**i, k**) and blast-exposed (**j, l**) animals. Panels (**i)** and (**j)** correspond approximately to coordinates interaural 12.24–9.48 mm and panels (**k)** and (**l)** correspond approximately to coordinates interaural 6.94–3.24 mm. Lateral views of (**i)** and **(j)** are shown in (**m)** and (**n)**, respectively. Vessels were color coded to allow visualization of individual vessels automatically traced by the Vesselucida 360 software. Note the general loss of radial organization in the blast-exposed shown in panel (**j)**. Scale bar, 1 mm for (**i**-**n**)

To determine whether blast induces chronic vascular pathology, we obtained high-resolution X-ray micro-CT scans of the brain vasculature of rats at 10 months after blast exposure following perfusion with the Brite Vu contrast agent. Figure 16 shows maximum intensity projection (MIP) images of the volume-rendered brain vasculature from the brains of 2 control and 2 blast-exposed animals reconstructed with Bruker’s CTVox 3D visualization software. A generalized thinning of the cerebral vasculature is noticeable in the blast-exposed animals (Fig. 16a-d). Panels e-p in Fig. 16 show visualization of the micro-CT data reconstructed for quantitation with the software Vesselucida. Quantitative analyses of these data showed a blast-induced decrease in total brain vascular length (about 50%; 6.22 × 10^6^ ± 1.31 × 10^6^ μm for control vs 3.16 × 10^6^ ± 2.06 × 10^4^ μm for blast), total surface area (over 50%; 9.99 × 10^8^ ± 1.55 × 10^8^ μm^2^ for control vs 4.68 × 10^8^ ± 1.88 × 10^7^μm^2^ for blast) and total volume (almost 60%; 1.52 × 10^10^ ± 1.17 × 10^9^ μm^3^ for control vs 0.62 × 10^10^ ± 5.17 × 10^8^ μm^3^). Imaging analyses also revealed a general loss of vascular organization with disruption of the regular radial patterns visible in control brain (Fig. 14i-j). Thus, despite recovery of GFAP and NFH associated with the vascular fraction, a chronic blast-induced vascular pathology remained as evidenced by micro-CT scanning, a finding consistent with prior work demonstrating chronic vascular pathology in this model between 6 and 10 months following blast exposure [31].

## Discussion

We have been studying a rat model developed to mimic blast exposures that would be associated with human mTBI or a subclinical exposure, an exposure that we refer to as “low-level” [29]. In multiple studies the lack of generalized neuronal pathology at the light and EM levels has been confirmed as well as the absence of any generalized reactive astrocytosis or inflammatory reaction [2, 22, 26, 31–33]. Because multiple blast exposures have been common among veterans returning from Iraq and Afghanistan [28], for most studies including the present we used a design in which rats received three exposures delivered one per day on 3 consecutive days. Rats exposed to these blast conditions develop a variety of chronic PTSD-related behavioral traits [26, 64–67].

Despite the generally benign appearance of the brain in standard histopathology at low magnification, at the EM level, a prominent vascular pathology was visible [31]. To explore blast-induced molecular changes in the vasculature we conducted proteomic studies using purified brain vascular fractions. The present study stems from the unexpected observation that GFAP was decreased in isolated brain vascular fractions from blast-exposed animals (6 weeks after exposure). These findings were confirmed by quantitative Western blotting on a separate cohort of animals harvested also 6 weeks post-blast exposure.

Many studies have documented astrogliosis following higher level blast exposures [27], and a distinct type of glial scar has been described in humans exposed to blast injury [79]. By contrast we found a decrease of GFAP in isolated brain vascular fractions from blast-exposed animals. In a regional analysis, GFAP was either normal or decreased in selected brain regions further arguing against any generalized astrogliosis in this model. Differences in GFAP in isolated vascular fractions did not seem to be accounted for by differences in the relative arterial vs. venous compositions of the vascular fractions isolated from control and blast-exposed brains as no differences were noted in Western blotting with antibodies against a preferential marker of arterial smooth muscle (α-SMA) or a marker of venous endothelial cells (EPHB4) [24]. Micro CT findings suggested a thinning of the brain vasculature following blast exposure. Because in the 2D-DIGE and Western immunoblotting analyses equal amounts of protein from blast-exposed and control vascular preparations were compared, the reduction in GFAP and neurofilament proteins cannot be explained by a loss of brain vasculature in blast-exposed animals. Loss of GFAP in isolated vascular fractions suggested a widespread loss of gliovascular connections following blast, a speculation confirmed with GFAP immunostaining of isolated vascular fractions. Loss of gliovascular coverage was not clearly visible in sections immunostained for GFAP and collagen IV. However, EM studies revealed swollen and disrupted astrocytic endfeet around small cortical vessels, suggesting that reduced GFAP levels previously observed in isolated vascular fractions may have resulted from loosened gliovascular connections (due to astrocytic degeneration) that were lost during the isolation procedure. Equally surprising in the initial proteomic study was a loss of a series of neuronal IF proteins which suggested widespread loss of neurovascular connections as well.

Although loss of GFAP and neuronal IF proteins associated with the vasculature was reversed by 8 months after blast exposure, a chronic vascular pathology was still visible histologically and by micro-CT scanning. These findings suggest that normalization of GFAP and IF protein levels in isolated vascular fractions, while likely signaling the tightening of gliovascular and neurovascular connections, does not translate into a resolution of the vascular pathology. Chronic vascular alterations induced by blast exposures have been previously documented in this model at 6–10 months following blast exposure [31].

Vascular pathology is a well-described feature of blast-related brain injury in both animals and humans [1, 7, 18, 19, 27, 31, 32, 37, 42, 46–48, 51–53, 55, 58, 61, 68, 69, 72, 73, 76, 80, 84, 87] with multiple studies describing increases in BBB permeability [1, 36, 45, 49, 52, 54, 56, 57, 60, 61, 71, 74, 81, 85, 90, 92, 93]. What is novel about the present studies is the suggestion that the basis for these changes is at least in part the result of a widespread disruption of gliovascular and neurovascular connections in a model that lacks any obvious diffuse neuronal structural pathology. The fact that these changes were apparent in Western blots of vascular fractions obtained from whole brain further supports the widespread nature of the changes.

Several prior studies have described ultrastructural changes in astrocytic endfeet following blast exposure [37, 46, 55]. In a rat model of blast injury, Kaur et al. [46] observed that astrocytes in the cerebral and cerebellar cortex were hypertrophied with swollen endfeet that were sparsely populated with organelles. However, this pathology appeared only transiently and was absent in rats studed 14–28 days after blast exposure. Lu et al. [55] described hypertrophied and “watery” astrocytic endfeet in a non-human primate model utilizing live explosives. These changes however occurred in the context of a broader neuronal, glial and microglial pathology that was absent in our model. Goldstein et al. [37] described swollen astroyctic endfeet in a mouse model of blast injury. This pathology however also occured in the setting of a broader neuropathology with neuronal tau pathology, axonal changes and widespread astrogliosis. In addition in these studies when the head was immobilized hippocampal learning and memory deficits were no longer apparent arguing that effects were occurring primarily on the basis of acceleration-deceleration/rotational injury and not as a primary effect of blast, unlike in our studies in which the head was immobilized and no broader neuropathology was apparent.

Several limitations of this study must be mentioned. Firstly the time course of the progression of gliovascular and neurovascular changes has not been defined. Because of our interest in the aspects of blast exposure most relevant to the veteran population we have focused on blast’s effects on the subacute (6 weeks) to chronic (8–10 month) time periods after exposure. In a previous study we described acute vascular pathology 24–72 h after blast exposure [31]. However, these studies will need to be expanded into a broader longitudinal study that will include ultrastructural analysis of samples from the acute to chronic phases. While the levels of vascular-associated GFAP recover in Western blots to control levels at 8 months post-exposure, we do not know whether this reflects recovery of normal gliovascular connections. In addition, the micro CT results further support the widespread nature and chronicity of the vascular degenerative processes in this model in a manner that is more easily appreciated than in tissue sections. However, it will be necessary to expand the sample size and time points for the micro CT studies in order to capture the full evolution of the disease process.

It should also be noted that we have not explored the full extent of the molecular changes in blast-exposed vessels. The 2D-DIGE system by its nature only detects differences in relatively high abundance proteins. There are likely many other changes that went undetected in our screen. Others have reported changes in the BBB tight-junction proteins occludin, claudin-5, and zonula occludens 1 (ZO-1) as well as in VE-cadherin, Chemokine (C-C Motif) Ligand 2 (CCL2) and VEGF following blast exposure [1, 3, 54, 57, 75, 89] and to date we have only identified slightly more than a dozen of the 77 spots that differed between control and blast-exposed in the initial screen.

Because gliovascular and neurovascular interactions control cerebral blood flow at multiple levels, their disruption following blast exposure would be expected to affect cerebral autoregulation. Astrocytes play important roles in mediating neurovascular coupling (reviewed in [43]). Glutamate released from neurons acts on astrocytic metabotropic glutamate receptors to evoke Ca^2+^-dependent release of vasoactive metabolites from astrocytic endfeet. Under normoxic conditions, astrocytic Ca^2+^ signaling results in vasodilation, whereas under hyperoxia, vasoconstriction is favored.

Vascular radius is the most powerful determinant of blood flow, with even small changes in lumen diameter having significant effects on cerebral blood flow [63]. Cerebral pial vessels are innervated in the adventitial layer by perivascular nerves originating from both autonomic and sensory ganglia of sympathetic, parasympathetic and trigeminal origin (reviewed in [40]). Sympathetic terminals have significant vasoconstrictor effects in large cerebral arteries. Under conditions of elevated hydrostatic pressure, sympathetic vasoconstriction protects against increases in venous pressure, disruption of the BBB and edema formation. Stimulation of parasympathetic nerves has potent vasodilator effects on cerebral arteries, resulting in increased blood flow. Additional intrinsic cerebral hemodynamic regulatory mechanisms exist, as sympathetically and parasympathetically denervated animals exhibit cerebral blood flow autoregulation [11]. Thus altered neurovascular connections could disrupt cerebral autoregulation at multiple levels.

In addition to disrupted gliovascular and neurovascular signaling, the cerebral circulation could be also impaired by alterations in vascular smooth muscle. As demonstrated here, blast exposure results in structural alterations of the arterial medial smooth muscle layer. Interestingly, blast-induced vasospasm has been suggested to initiate a phenotypic switch in vascular smooth muscle cells that causes long-term vascular remodeling [4, 39]. Whether the changes observed here are part of such a switch will require further study.

Moreover, in some small cortical and hippocampal arterial vessels, lumenal occlusion by cells expressing CD34 (and sometimes GFAP) was observed along with depletion of the CD34^+^ cells in the adventitia. CD34-expressing cells are a well-characterized population of stem cells that have the capacity to reconstitute the hematopoietic system [59]. In the arterial adventitia, these cells are likely remnants of earlier developmental stages and may participate in repair processes, as they are capable of differentiating into different vascular cell types, including smooth muscle cells, pericyte-like cells, CD34-expressing angiogenic progenitors and macrophages [62, 82]. The concomitant depletion of arterial adventitial CD34-expressing cells together with the partial arterial occlusion they cause argues for the migration of these cells from the adventitia into the lumen as a step possibly related to vascular repair mechanisms.

## Conclusion

Our results show that blast exposure affects cellular interactions within the neurovascular unit including loss of gliovascular and neurovascular interactions as well as smooth muscle degeneration. The cognitive and other behavioral alterations that develop following blast exposure in both humans and animal models [25, 26, 28, 29, 64–66] may in turn be caused by defective circulatory effects resulting from disrupted neurogliovascular cellular communication.
